# Gaining or cutting SLAC: the evolution of plant guard cell signalling pathways

**DOI:** 10.1111/nph.20172

**Published:** 2024-10-06

**Authors:** Frances C. Sussmilch, Tobias Maierhofer, Johannes Herrmann, Lena J. Voss, Christof Lind, Maxim Messerer, Heike M. Müller, Maria S. Bünner, Peter Ache, Klaus F. X. Mayer, Dirk Becker, M. Rob G. Roelfsema, Dietmar Geiger, Jörg Schultz, Rainer Hedrich

**Affiliations:** ^1^ Molecular Plant Physiology and Biophysics University of Würzburg Julius‐von‐Sachs Platz 2 Würzburg D‐97082 Germany; ^2^ School of Natural Sciences University of Tasmania Private Bag 55 Hobart 7001 TAS Australia; ^3^ Plant Genome and Systems Biology Helmholtz Center Munich Ingolstädter Landstraße 1 Neuherberg 85764 Germany; ^4^ Department of Bioinformatics, Biozentrum University of Würzburg, Am Hubland Klara‐Oppenheimer‐Weg 32, Campus Hubland Nord Würzburg D‐97074 Germany; ^5^ Center for Computational and Theoretical Biology University of Würzburg Klara‐Oppenheimer‐Weg 32, Campus Hubland Nord Würzburg D‐97074 Germany; ^6^ School of Life Sciences Weihenstephan Technical University of Munich Alte Akademie 8 Freising 85354 Germany; ^7^ College of Science King Saud University PO Box 2455 Riyadh 11451 Saudi Arabia

**Keywords:** abscisic acid, angiosperms, ferns, OPEN STOMATA 1, plant evolution, seed plants, stomata, S‐type anion channel (SLAC/SLAH) family

## Abstract

- The evolution of adjustable stomatal pores, enabling CO_2_ acquisition, was one of the most significant events in the development of life on land. Here, we investigate how the guard cell signalling pathways that regulate stomatal movements evolved.
- We compare fern and angiosperm guard cell transcriptomes and physiological responses, and examine the functionality of ion channels from diverse plant species.
- We find that, despite conserved expression in guard cells, fern anion channels from the SLAC/SLAH family are not activated by the same abscisic acid (ABA) pathways that provoke stomatal closure in angiosperms. Accordingly, we find an insensitivity of fern stomata to ABA. Moreover, our analysis points to a complex evolutionary history, featuring multiple gains and/or losses of SLAC activation mechanisms, as these channels were recruited to a role in stomatal closure.
- Our results show that the guard cells of flowering and nonflowering plants share similar core features, with lineage‐specific and ecological niche‐related adaptations, likely underlying differences in behaviour.

## Introduction

Land plants evolved from a green algal ancestor, which conquered dry land over 500 million years ago (Morris *et al*., 2018). The adjustable stomatal pore, which enables CO_2_ acquisition in cuticle‐covered tissues, is a key innovation that likely helped plants to thrive on land. The stomatal pore is usually flanked by two guard cells that regulate stomatal aperture, found in all major land plant lineages except liverworts (see Duckett & Pressel, 2018). In vascular plants (lycophytes, ferns, gymnosperms and angiosperms), mature stomata are able to close and reopen in response to internal and environmental signals and have an important role in preventing excessive water loss. In contrast to those of vascular plants, stomata in mosses and hornworts appear important for drying spores and sporophyte dehiscence (Duckett *et al*., 2009; Chater *et al*., 2016; Renzaglia *et al*., 2017). Moss stomata may respond to some stimuli during early development, including exogenous ABA application (Chater *et al*., 2011), but develop mechanical restrictions that prevent closure at maturity (Merced & Renzaglia, 2014; Duckett & Pressel, 2022). Mosses in the genus *Sphagnum* have pseudostomata, which lack a stomatal pore but possess two guard cells that collapse irreversibly, promoting spore desiccation (Duckett *et al*., 2009; Merced, 2015). Hornwort stomata open once only through irreversible guard cell collapse (Renzaglia *et al*., 2017).

Stomata have been most thoroughly studied in angiosperm species, which have revealed the specialised multisensory signalling pathways, cell wall and membrane components that enable guard cells to undergo rapid directed volume changes for active stomatal movements. Stomatal closure in response to dehydration stress is mediated by the hormone abscisic acid (ABA), synthesised in leaves and even guard cells themselves during drought stress (e.g. Mittelheuser & Van Steveninck, 1969; Bauer *et al*., 2013; McAdam *et al*., 2016b). In diverse angiosperms, orthologs of the S‐type anion efflux channel SLOW ANION CHANNEL 1 (SLAC1) are activated upon phosphorylation by the serine/threonine protein kinase OPEN STOMATA 1 (OST1) from the SNF1‐related protein kinase 2 (SnRK2) family, in an ABA‐dependent, calcium‐independent manner (Vahisalu *et al*., 2008; Geiger *et al*., 2009; Müller *et al*., 2017; Y. Li *et al*., 2022). In addition, SLAC1 and a member of the same ion channel family – SLAC1 HOMOLOGUE 3 (SLAH3) – are sensitive to calcium signals via activation by calcium‐dependent protein kinases (CPKs) and/or calcineurin B‐like protein (CBL)‐interacting protein kinases (CIPKs) (Geiger *et al*., 2010, 2011; Scherzer *et al*., 2012; Maierhofer *et al*., 2014a; Huang *et al*., 2023). ABA can also induce calcium signals in guard cells before stomatal closure (McAinsh *et al*., 1990), and the CPKs and CIPKs form an OST1‐independent branch of the ABA‐signalling pathway (Mori *et al*., 2006; Geiger *et al*., 2010; Scherzer *et al*., 2012; Brandt *et al*., 2015). Other members of the SLAC/SLAH family – SLAH1, SLAH2 and SLAH4 – have apparent roles in translocation of Cl^−^ and NO_3_^−^ ions in roots and vascular tissues for nutrition and salinity tolerance (Maierhofer *et al*., 2014b; Cubero‐Font *et al*., 2016; Qiu *et al*., 2016).

Abscisic acid perception and signalling components appear largely conserved between land plants (e.g. Bowman *et al*., 2017; Xiao *et al*., 2018; Shinozawa *et al*., 2019; Sun *et al*., 2019; Nibau *et al*., 2022). However, it is currently debated whether or not ABA also has a conserved function in stomatal closure in seedless plant groups (see Sussmilch *et al*., 2019b; McAdam *et al*., 2021; Clark *et al*., 2022; Chater, 2024). There are numerous reports of stomatal responses to applied ABA in seedless lineages including mosses, lycophytes and ferns, dependent on conditions (Chater *et al*., 2011; Ruszala *et al*., 2011; Hõrak *et al*., 2017). Coupled with phylogenomic studies indicating gene losses in bryophytes, these findings have led to the popularity of the theory that land plant stomata had a complex, ABA‐sensitive ancestral state (e.g. Clark *et al*., 2022). In support of a more gradual evolution of ABA‐mediated stomatal control mechanisms, so far only seed plants have been shown capable of closing stomata in response to elevated endogenous ABA levels (Brodribb & McAdam, 2011; McAdam & Brodribb, 2012), coinciding with the evolutionary expansion of the gene families involved in ABA‐dependent stomatal closure (Bowles *et al*., 2022). Intriguingly, experiments in *Xenopus* oocytes have revealed that SLAC/SLAH channels from the alga *Klebsormidium nitens*, the liverwort *Marchantia polymorpha*, the lycophyte *Selaginella moellendorffii* (all SmSLAC homologues tested) and the fern *Ceratopteris richardii* (two CrSLAC homologues tested) are not activated by OST1 kinases from the respective species (McAdam *et al*., 2016a), in contrast to angiosperm SLAC1 channels (e.g. Geiger *et al*., 2009; Müller *et al*., 2017; Qi *et al*., 2018; Schäfer *et al*., 2018). However, a functional OST1‐SLAC pair was isolated from the moss *Physcomitrium/Physcomitrella patens*, indicating that at least one bryophyte also has a SLAC homologue that can be activated by an ABA‐sensitive kinase (Lind *et al*., 2015).

In this study, we investigated the evolution of guard cell signalling pathways. We performed RNA‐seq experiments to find out what makes guard cells special compared with other leaf cells in vascular plants, and what transcriptional features separate angiosperm and fern guard cells. For this, we used two fern species, *C. richardii* and *Polypodium vulgare*, in comparison with the angiosperms *Arabidopsis thaliana* and barley (*Hordeum vulgare*). *C. richardii* is an aquatic fern with a long history as a model for genetics (e.g. Hickok *et al*., 1987, 1995), and *P. vulgare* is an historical stomatal research model capable of rapid, reversible stomatal responses to air humidity (Lange *et al*., 1971; Stevens & Martin, 1977; Lösch, 1979). Our results indicate that these fern guard cells express homologues of many important angiosperm guard cell genes, including those encoding SLAC/SLAH ion channels and kinases from the SnRK2, CPK and CIPK families. We tested the activity of the guard cell‐expressed fern SLAC proteins and examined the evolution of SLAC activity in land plants using additional bryophyte and seed plant models. We found that fern SLAC proteins are not activated by the same ABA‐dependent pathways as angiosperms. Our results reveal the diversity of guard cell regulatory mechanisms in plants alive today, while shedding light on how these have evolved over the past 500 million years of plant life on land.

## Materials and Methods

### ABA electro‐infusion

*Polypodium vulgare* L. plants were obtained from the Würzburg Botanical Gardens and grown in a glasshouse with *Nicotiana tabacum* L. cv SR1, under natural light extended with HQL‐pressure lamps (Powerstar HQI‐E, 400 W; Philips, Eindhoven, the Netherlands) at a 12 h : 12 h, day : night cycle. Extracellular application experiments (current ejection) with microelectrodes were conducted with intact plants. The adaxial side of leaves was attached with double‐sided adhesive tape to a Plexiglas holder in the focal plane of an upright microscope (Axioskop 2FS; Zeiss). Stomata were visualised with a water immersion objective (W Plan‐Apochromat, 40×/0.8, or 63×/1.0; Zeiss) dipped into bath solution (5 mM KCl, 0.1 mM CaCl_2_ and 5 mM K‐citrate, pH 5.0) on the abaxial surface of the leaf. Microelectrodes were put in contact with the cell wall of stomata with a piezo‐driven micro‐manipulator (MM3A; Kleindiek Nanotechnik, Reutlingen, Germany), as described previously (Huang *et al*., 2019). The microelectrodes were prepared from borosilicate glass capillaries (inner diameter, 0.58 mm; outer diameter, 1.0 mm; Hilgenberg) and filled with solution containing 1 mM Lucifer Yellow (LY) and 1 mM ABA, or only 1 mM LY (control). The ABA concentration in the electrodes was 20 times higher than that used by Huang *et al*. (2019) and a −1 nA ejection current was applied, instead of −0.8 nA used by Huang *et al*. (2019). In comparison with the analysis of Huang *et al*. (2019), current ejection will have resulted in a local ABA concentration of 25 μM, which decreases by 50% over 1 μm.

The stomatal movements and ejection of LY were monitored with a charge‐multiplying CCD camera (QuantEM; Photometrics, Tucson, AZ, USA). The fluorescent probe was excited with a Hg metal‐halide lamp (HXP120; Leistungselektronic JENA, Jena, Germany), through a band pass filter of 430/24 nm (ET 430/24; Chroma Technology Corp., Bellows Falls, VT, USA). A dichroic mirror (495 nm LP) guided the excitation light through the objective, while the fluorescent light was filtered with an emission band pass filter (520/30 nm; BrightLine, Semrock, West Henrietta, NY, USA). The filters could be rapidly moved in and out of the light path, with filter wheels of a spinning disc confocal unit (CARV II; Crest Optics, Rome, Italy) that was mounted to the camera port of the upright microscope. Image acquisition was conducted with visiview software (Visitron, Puchheim, Germany) and analysed the image‐j/fiji software package (Schindelin *et al*., 2012).

### Arabidopsis RNA‐seq

*Arabidopsis thaliana* (L.) Heynh. plants were grown in semi‐sterilised soil (treated for 20 min at 100°C), cultivated in climate chambers (Binder KBWF 720) in a 12‐h day–night rhythm (22/16°C, 60% RH) and were illuminated with 125 μmol m^−2^ s^−1^ white light. RNA was isolated from whole leaf samples and guard cell‐enriched samples isolated by mechanical disruption as described previously (Bauer *et al*., 2013), from 6 to 7 wk old plants. Library preparation and RNA‐seq were carried out as described in the Illumina TruSeq Stranded mRNA Sample Preparation Guide, the Illumina NextSeq 500 System Guide (Illumina Inc., San Diego, CA, USA), and the KAPA Library Quantification Kit – Illumina/ABI Prism User Guide (Kapa Biosystems Inc., Woburn, MA, USA).

Briefly, 250 ng of total RNA was used for purifying the poly‐A‐containing mRNA molecules using poly‐T oligo‐attached magnetic beads. Following purification, the mRNA was fragmented to an average insert size of 200–400 bases using divalent cations under elevated temperature (94°C for 4 min). Next, the cleaved RNA fragments were reverse transcribed into first‐strand cDNA using reverse transcriptase and random hexamer primers. Actinomycin D was added to improve strand specificity by preventing spurious DNA‐dependent synthesis. Blunt‐ended second strand cDNA was synthesised using DNA Polymerase I, RNase H and dUTP nucleotides. The incorporation of dUTP, in place of dTTP, quenched the second strand during the later PCR amplification. The resulting cDNA fragments were adenylated at the 3′ ends, the indexing adapters were ligated and subsequently specific cDNA libraries were created by PCR enrichment. The libraries were quantified using the KAPA SYBR FAST ABI Prism Library Quantification Kit. Equimolar amounts of each library were sequenced on a NextSeq 500 instrument using two 150 Cycles High Output and one 150 Cycles Mid Output Kits with the single index, paired‐end (PE) run parameters. Image analysis and base calling resulted in .bcl files, which were converted into .fastq files with the bcl2fastq v.2.18 software. Library preparation and RNA‐seq were performed at the service facility ‘KFB – Center of Excellence for Fluorescent Bioanalytics’ (Regensburg, Germany).

### Barley RNA‐seq

Barley (*Hordeum vulgare* L. cv. Barke) seeds were provided by a commercial supplier (Saatzucht J. Breun GmbH & Co. KG, Herzogenaurach, Germany) and cultivated at 22/16°C and 50 ± 5% RH at a 12 h : 12 h, day : night cycle and a photon flux density of 500 mmol m^−2^ s^−1^ white light (400 W; Philips Master T Green Powers). For guard cell‐enriched samples, epidermal peels were first isolated from the abaxial side of 8‐ to 12‐d‐old leaves, and then, subsidiary cells were disrupted with successive blender cycles in ice‐cold water as described previously (Schäfer *et al*., 2018). RNA was extracted using the NucleoSpin® RNA Plant Kit (Macherey‐Nagel, Drueren, Germany). RNA isolation from whole leaves was performed similarly.

The extracted RNA was treated with RNase‐free DNase (New England Biolabs, Ipswich, MA, USA). Quality control measurements were performed on a 2100 Bioanalyzer (Agilent, Santa Clara, CA, USA) and the concentration was determined using a Nanodrop ND‐1000 spectrophotometer (Thermo Fisher Scientific, Wilmington, DE, USA). Libraries were prepared with the TruSeq RNA Sample Prep Kit v2 (Illumina Inc.) using 1 mg of RNA and sequenced on a HiSeq 3000 (Illumina Inc.) resulting in a sequence depth of 35 million PE reads (2× 150 bp).

### Fern RNA‐seq

*Ceratopteris richardii* Brongn. wild‐type (WT) strain Hn‐n (Hickok *et al*., 1995) was grown in controlled‐condition glasshouse facilities under a 16‐h photoperiod with supplemented natural light. *P. vulgare* plants (Common Polypody – winter hardy; Westdijk's, Kwekerijen) were grown in a growth chamber under a 12 h photoperiod, with 22°C : 16°C, day : night temperatures.

Four biological replicates, each comprising up to 100 mg tissues, were collected from both species for whole leaf samples comprising fully expanded sporophyte fronds lacking sporangia and guard cell‐enriched samples. Guard cell‐enriched samples were obtained from fully expanded fronds lacking sporangia, after removal of the main veins, by mechanical disruption of other cell types using successive 1–2 min blending cycles in deionised ice water with epidermal fragments collected by filtration through a 210 μm nylon mesh, according to a previously published method (Bauer *et al*., 2013) optimised for *C. richardii* (3 cycles) and *P. vulgare* (5 cycles). Fluorescein diacetate staining (Widholm, 1972) was used to confirm guard cell viability and purity. In *P. vulgare*, ‘leaf without epidermis’ samples lacking guard cells were also obtained by using fine forceps to fully remove the abaxial epidermis from leaves.

Total RNA was extracted, treated with RNase‐free DNase and quality control measurements were performed as described above. Library preparation and mRNA‐seq after polyA‐enrichment was performed by the Core Unit Systems Medicine (University of Würzburg). Both species were sequenced on the Illumina NextSeq500 platform using a single high‐output flow cell for the 8 *C. richardii* samples and duplicate high‐output flow cells for the 12 *P. vulgare* samples for 150 nt PE reads (300 cycles).

### Transcriptome assembly and annotation

Transcriptomes for *P. vulgare* and *C. richardii* were assembled using trinity (Grabherr *et al*., 2011) with the trimmomatic option (Table S1). Coding regions were predicted with TransDecoder (https://github.com/TransDecoder/TransDecoder). Domains and GeneOntology annotations were predicted using InterPro (Blum *et al*., 2021). To generate a level of annotation comparable to the fern data, all proteomes (Table S2) were annotated *de novo* using InterPro.

### Differential expression

For the ferns, reads were mapped onto the reference transcriptomes by Salmon (Patro *et al*., 2017). For *A. thaliana* and *H. vulgare*, the reads were mapped onto the reference genomes (Table S2) by RSEM (Li & Dewey, 2011). Differentially expressed genes were identified with deseq2 (Love *et al*., 2014).

### Evolutionary reconstruction

Orthology relationships over all species (Table S2) were predicted using Orthofinder (Emms & Kelly, 2015). The last common ancestor for each orthogroup was calculated using the Bio::TreeIO perl package. Expression groupings were classified into hierarchical categories of genes ‘up’‐regulated (1) expression higher in guard cells than leaves AND higher in whole leaves than leaves without guard cells – *P. vulgare* only, *P*adj ≤ 0.01; (2) expression higher in guard cells than leaves, *P*adj ≤ 0.01; (3) expressed in guard cell samples, baseMean ≥ 10; (4) expression negligible in guard cell samples, baseMean < 10 or ‘down’‐regulated (1) expression lower in guard cells than leaves AND lower in whole leaves than leaves without guard cells – *P. vulgare* only, *P*adj ≤ 0.01; (2) expression lower in guard cells than leaves, *P*adj ≤ 0.01 in guard cells relative to whole leaf samples based on the presence of one or more genes in the orthogroup meeting the category criteria (Table S3).

### Phylogenetic analyses for streptophyte SLAC/SLAH and SnRK2 families

Genes were identified in the literature or by performing BLASTp searches using Arabidopsis protein sequences against the relevant genome or transcriptome assembly as indicated in Table S4, and confirmed with reciprocal BLASTp searches back against Arabidopsis and preliminary phylogenetic analyses. The maximum likelihood phylogenetic tree for the SLAC/SLAH family was calculated from a MAFFT alignment of full‐length predicted protein sequences using phyml v.3.0 at http://www.trex.uqam.ca/ with the JTT substitution model and 1000 bootstrap replicates (Guindon *et al*., 2010). The maximum likelihood phylogenetic tree for the SnRK2 family was calculated using phyml v.3.0 at http://www.atgc‐montpellier.fr/phyml/ with SmartModel Selection (Guindon *et al*., 2010; Lefort *et al*., 2017), and 1000 bootstrap replicates, from a MAFFT alignment of predicted protein sequences trimmed using Gblocks via the online server at http://molevol.cmima.csic.es/castresana/Gblocks_server.html (Talavera & Castresana, 2007), with all options for reduced stringency selected. Full sequence and species details are given in Table S4.

### Cloning and complementary RNA generation

Full‐length coding sequence from *OST1*, *SLAC* and/or *CPK* homologues of *C. richardii* (Hn‐n, obtained from J. Banks; leaf), *P. vulgare* (Common Polypody – winter hardy; Westdijk's, Kwekerijen; leaf), *Picea abies* (L) H. Karst. (plants maintained at the University of Tasmania; needles), *Ginkgo biloba* L. (plants maintained at the Würzburg Botanical Gardens; leaves), *Amborella trichopoda* Baill. (plants maintained at the University of Tasmania; leaves), *Sphagnum fallax* (H.Klinggr.) H. Klinggr. (‘MN’, obtained from S. Rensing, from an original sample from Dave Weston, Oak Ridge National Laboratory; gametophyte) and *Anthoceros agrestis* Paton (‘Bonn’, obtained from P. Szoevenyi; gametophyte) were isolated from cDNA generated from RNA from the tissues indicated using primers outlined in Table S5 and cloned into pNB1uYN and pNB1uYC expression vectors by uracil excision‐based cloning (Nour‐Eldin *et al*., 2006). The details for all *A. thaliana* constructs, and *C. richardii CrSLAC1a*, *CrSLAC1b*, *CrGAIA1* and *CrPGAI* constructs have been published previously (Geiger *et al*., 2009, 2010, 2011; Scherzer *et al*., 2012; Maierhofer *et al*., 2014a; McAdam *et al*., 2016a). Where indicated, site‐directed mutations were introduced using a modified USER fusion method, as previously described (Dadacz‐Narloch *et al*., 2011), using the primers outlined in Table S5. For functional analysis, complementary RNA (cRNA) was prepared with the AmpliCap‐Max™ T7 High Yield Message Maker Kit (Epicentre Biotechnologies). Oocyte preparation and cRNA injection were performed as described previously (Becker *et al*., 1996). For oocyte bimolecular Fluorescence Complementation (BiFC) and electrophysiological experiments, 10 ng of each SLAC:YFP^CT^ (vector pNB1uYC) and 10 ng of each OST1:YFP^NT^ or 5 ng of CPK:YFP^NT^ or AtCIPK23∆EF:YFP^NT^ + AtCBL1:YFP^NT^ (vector pNB1uYN) cRNA or cRNA of the same genes cloned into the pNB1u vector without YFP fragments, were injected into *Xenopus laevis* (Daudin) oocytes.

### Bimolecular fluorescence complementation experiments

Expression of BiFC constructs in oocytes was performed as described previously (Geiger *et al*., 2009). Images were taken with a confocal laser scanning microscope (Leica DM6000 CS; Leica Microsystems CMS GmbH, Wetzlar, Germany) equipped with a Leica HCX IRAPO L25×/0.95 W objective. Images were processed (low pass–filtered and sharpened) identically with the image acquisition software LAS AF (Leica Microsystems CMS GmbH).

### Double‐electrode voltage‐clamp studies

Oocytes were perfused with MES/Tris‐based buffers containing 10 mM MES/Tris (pH 5.6), 1 mM Ca(gluconate)_2_, 1 mM Mg(gluconate)_2_, 1 mM LaCl_3_ and 100 mM NaCl, NaNO_3_ or Na(gluconate). To balance ionic strength, changes in chloride or nitrate concentration were compensated with Na(gluconate). The standard voltage protocol was as follows: starting from a holding potential (V_H_) of 0 mV, single 50 ms‐voltage pulses were applied in 10 mV decrements from +70 to −150 mV, with instantaneous currents extracted right after the voltage jump from the holding potential.

## Results

### Fern and angiosperms share conserved guard cell expression of core genes but show lineage‐specific expression patterns for others

**Fig. 1:**
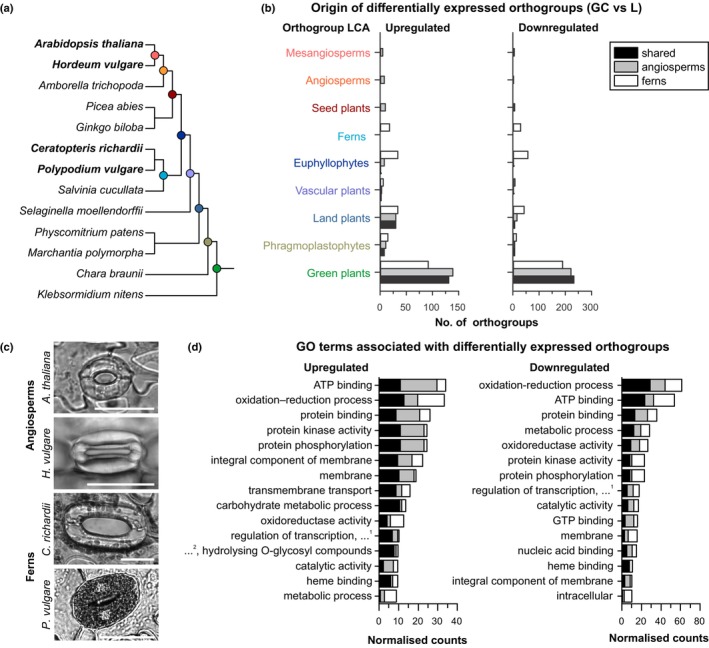
There are core sets of genes shared between angiosperm (*Arabidopsis thaliana*, *Hordeum vulgare*) and fern (*Ceratopteris richardii*, *Polypodium vulgare*) guard cell transcriptomes with some lineage‐specific differences. (a) Plant species included in RNA‐seq experiments (bold) and orthology analyses, with phylogenetic relationships indicated (branch lengths not to scale). (b) Last common ancestor (LCA) nodes of orthogroups with significantly different expression between guard cell (GC) and whole leaf (L) samples in all angiosperm and fern species examined (‘shared’; black), both angiosperm but neither fern species (‘angiosperms’; grey), and both fern but neither angiosperm (‘ferns’; white). (c) Photographs of guard cells of the species included in RNA‐seq experiments (bar, 50 μm). (d) The top 15 most common Gene Ontology (GO) terms of orthogroups up‐ or downregulated in guard cell relative to whole leaf samples, with counts normalised to the total number of genes per orthogroup. Abbreviations are as follows: …^1^ – ‘DNA templated’, …^2^ – ‘hydrolase activity’. See Supporting Information Fig. S1 for the most common annotated domains and Table S3 for all GO terms/annotated domains related to these orthogroups.

For insight into the transcriptional features of fern guard cells compared with those of angiosperms, we performed RNA‐seq experiments using Arabidopsis, barley, *C. richardii* and *P. vulgare*. For each species, we enriched guard cells by mechanical isolation of epidermal fragments through disruption of other cell types (Bauer *et al*., 2013) and identified genes significantly (*P* ≤ 0.01) up‐ or downregulated in guard cells relative to whole leaves. To reconstruct the evolutionary history of these genes, we extended our species set with representative models from major land plant lineages and streptophyte algae. Prediction of orthogroups based on this diverse dataset allowed us to identify genes derived from a single common ancestor. We classified three types of orthogroups of interest containing genes up‐ or downregulated in guard cell samples: (1) shared (genes differentially expressed in all four species), (2) angiosperm‐specific (Arabidopsis and barley only) and (3) fern‐specific (*C. richardii* and *P. vulgare* only). We found that the majority of these differentially expressed orthogroups included green algal genes, suggesting an ancient evolutionary origin and likely presence in an algal ancestor of land plants before terrestrialisation, predating specialisation in different embryophyte cell types including guard cells (Fig. 1a–c).

To gain insight into the function of the orthogroups differentially expressed in guard cells, we compared domain content and Gene Ontology (GO) classifications. We found that guard cells of all four vascular plant species shared expression of genes associated with ATP‐binding, protein kinase activity/phosphorylation and membrane components (Figs 1d, S1; Table S3). Specific examples of shared guard cell orthogroups included *POLYGALACTURONASE INVOLVED IN EXPANSION* (*PGX*) family genes associated with pectin degradation and guard cell wall mechanics (Rui *et al*., 2017; Yi *et al*., 2018), and aquaporins from the plasma intrinsic proteins (PIP) family, which transport water and other solutes including CO_2_ across the plasma membrane (Bienert *et al*., 2018).

Angiosperm‐specific guard cell orthogroups were frequently classified by GO terms associated with energy transfer, signalling and/or membrane components (Fig. 1d; Table S3). This is in line with further specialisation for these types of genes in angiosperms, where stomatal movements are tightly regulated by complex signalling pathways (see Sussmilch *et al*., 2019b). Examples of angiosperm‐specific guard cell orthogroups included cellulose synthase/cellulose synthase‐like (CESA/CSL) superfamily genes, and C‐type lectin receptor‐like kinase genes linked to immune responses (Sun *et al*., 2020).

Conversely in fern guard cells, genes associated with protein kinase activity and phosphorylation were commonly downregulated (Figs 1d, S1; Table S3), including genes from the Cytosolic ABA Receptor Kinase (CARK) family involved in ABA signalling in Arabidopsis (see L. Zhang *et al*., 2018; X. Li *et al*., 2022). Instead, orthogroups associated with oxidation–reduction and metabolic processes were over‐represented in ferns, including orthologs of Arabidopsis mitochondrial glutamate dehydrogenase genes *GDH1/2/3* which feed the tricarboxylic acid (TCA) cycle to release stored energy (Fontaine *et al*., 2012), and phosphoglucomutases *PGM1/2/3* involved in starch/sucrose/cell wall synthesis (Malinova *et al*., 2014). This may reflect the higher abundance of chloroplasts in fern guard cells (Fig. 1c; Voss *et al*., 2018), which work together with mitochondria to supply cells with energy and metabolites.

### Fern guard cells express homologues of ABA biosynthesis and signalling genes

Genetic signalling pathways controlling stomatal movements have been well described in angiosperms, but the extent to which these are conserved in guard cells of other plant lineages is less well understood. Using our angiosperm and fern guard cell RNA‐seq data, we looked specifically at homologues of key angiosperm genes to identify those that are expressed and/or upregulated in guard cell relative to whole leaf samples. For *P. vulgare* – which has stomata limited to the abaxial leaf surface – we also compared whole leaf samples to leaf samples with abaxial epidermis removed (lacking guard cells), to identify which genes were expressed at both (1) a higher level in guard cells than whole leaves and (2) a higher level in whole leaves than leaf samples lacking guard cells.

**Fig. 2:**
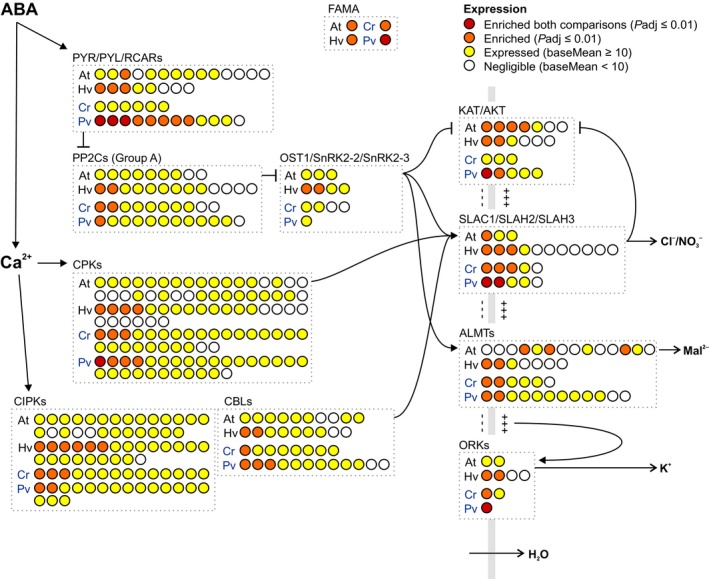
Homologues of key abscisic acid (ABA)‐signalling genes and ion channels are expressed in both angiosperm and fern guard cells. Groups of related genes are shown together in boxes with each circle representing a different gene, and colour coding representing relative expression in guard cell‐enriched samples compared with whole leaves shown for angiosperm (At, *Arabidopsis thaliana*; Hv, *Hordeum vulgare*, barley) and fern (Cr, *Ceratopteris richardii*; Pv, *Polypodium vulgare*) models, as indicated. For *P. vulgare* samples only, whole leaf vs leaf samples without abaxial epidermis (thus guard cells removed) were also included and used to separate guard cell‐enriched genes with a higher level of stringency (red; ‘enriched both comparisons’ = expression higher in guard cells than leaves and higher in whole leaves than leaves without guard cells). Homologues of the guard cell specification gene *FAMA* are also shown. The vertical grey line represents the guard cell plasma membrane with guard cell cytoplasm on the left and apoplast on the right. The role of gene products in guard cell signalling is indicated for key Arabidopsis genes (closed arrowhead, activation; blunt‐ended line, deactivation; open arrowhead direction of ion/water flow); please note that this may not reflect the characteristics of all homologues. See Supporting Information Fig. S2 for ABA biosynthesis genes and Table S6 for details for all genes of interest examined.

Consistent with angiosperms, homologues of the conserved guard cell specification gene *FAMA* (Ohashi‐Ito & Bergmann, 2006; Chater *et al*., 2016), showed significantly higher expression in fern guard cells than whole leaf samples (Fig. 2; Table S6). Similarly, both ferns showed expression of homologues of ABA biosynthesis and signalling genes in the guard cell samples, similar to the angiosperms (Figs 2, S2; Table S6). Although homologues of ABA2 – a short chain dehydrogenase (SDR) dedicated to the ABA biosynthesis pathway (Cheng *et al*., 2002; González‐Guzmán *et al*., 2002) – are restricted to angiosperms (Moummou *et al*., 2012; McAdam *et al*., 2015; Sussmilch *et al*., 2017), other SDRs are likely able to catalyse this step, and some closely related SDRs were expressed in the fern guard cell samples (Fig. S2; Table S6). Among the ABA‐signalling pathway, some genes encoding phosphatases from protein phosphatase type 2C clade A (PP2CA) and kinases from the CPK and CBL‐interacting kinase (CIPK) families were expressed at higher levels in guard cell than whole leaf samples in the ferns (Fig. 2). Other ABA‐signalling genes including members of the *OST1* subclade of the *Sucrose Non‐fermenting‐1‐Related Protein Kinase 2* (*SnRK2*) family were also nonspecifically expressed in the guard cells of the ferns, indicating their presence but likely nonspecific role in guard cells. Among the ion channels that are downstream of ABA‐signalling genes in angiosperms, S‐type anion channel genes from the SLAC/SLAH family were enriched in guard cells relative to whole leaves in all species. Overall, these results suggest that, similar to Arabidopsis and barley (Bauer *et al*., 2013; Schäfer *et al*., 2018), fern guard cells are equipped with the genetic toolkit required for ABA biosynthesis and signalling.

### Fern SLAC homologues are not activated by ABA‐signalling kinases

**Fig. 3:**
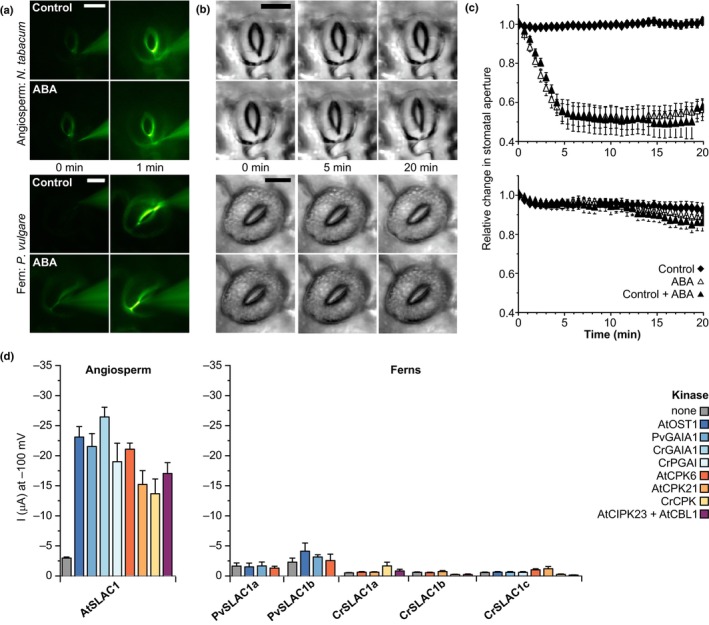
Unlike angiosperm stomata, fern stomata are insensitive to abscisic acid (ABA), and fern guard cell SLOW ANION CHANNEL (SLAC) homologues are not activated by ABA‐signalling kinases. (a) Fluorescent images of guard cells of the angiosperm *Nicotiana tabacum* (upper panels) and fern *Polypodium vulgare* (lower panels) at the start and 1 min after current ejection of 1 mM Lucifer Yellow (LY; control), or 1 mM LY and 1 mM ABA (bar, 10 μm). (b) Brightfield images of the guard cells shown in (a) up to 20 min after current ejection. (c) Normalised stomatal aperture, plotted against time for *N. tabacum* (upper graph) and *P. vulgare* (lower graph). Current ejection was performed at *t* = 0. Stomata treated with LY (control, diamonds) and subsequently to ABA (control + ABA, closed triangles), or to ABA only (ABA, open triangles; mean ± SEM, *n* ≥ 7). (d) Mean whole‐oocyte current measurements at −100 mV in nitrate‐based solution with SLAC homologues from the angiosperm *Arabidopsis thaliana* and the ferns *P. vulgare* and *Ceratopteris richardii*, co‐expressed with or without the indicated kinases in *Xenopus* oocytes (mean ± SEM, *n* ≥ 4).

It is currently debated whether or not ABA has a role in stomatal closure in seedless plant groups including ferns (see Sussmilch *et al*., 2019b; McAdam *et al*., 2021; Clark *et al*., 2022). To examine the stomatal responses of the fern *P. vulgare* to ABA, relative to those of the angiosperm tobacco (*N. tabacum*), we adapted a current‐ejection method (Huang *et al*., 2019) to apply ABA directly to guard cell walls in intact fern plants and monitor stomatal movements with an upright fluorescence microscope. Microelectrodes, filled with ABA and/or LY, were put in contact with the apoplast, close to an open stoma. Application of a − 1 nA current for 1 min caused the appearance of fluorescence in guard cell walls of *P. vulgare* and tobacco (Fig. 3a), showing that negatively charged molecules can be applied to guard cells with this approach. The current ejection of LY into the guard cell walls did not provoke stomatal closure, while ABA provoked rapid stomatal closure in tobacco stomata (Fig. 3b,c). Based on the data in Huang *et al*. (2019), this procedure is estimated to result in a local concentration of 25 μM ABA at the tip of the electrode, which was in contact with the guard cell wall, with ABA concentration likely decreasing by 50% over *c*. 1 μm. To verify that stomata prechallenged with the control LY solution had not lost ABA responsiveness, we applied the stress hormone in a second ejection. ABA‐induced stomatal closure was similar between guard cells pretreated with LY and those that were not (Fig. 3c). This confirmed that apoplastic application of ABA with microelectrodes causes rapid closure of tobacco stomata, just as previously reported for Arabidopsis (Huang *et al*., 2019). In contrast with tobacco, no response to ABA could be observed for *P. vulgare* (Fig. 3b,c). These results indicate that, unlike angiosperms, stomatal response in the fern *P. vulgare* is not sensitive to ABA. These findings are consistent with those of others who have found a lack of stomatal responsiveness to ABA in ferns grown in glasshouse conditions (Brodribb & McAdam, 2011; McAdam & Brodribb, 2012; Cândido‐Sobrinho *et al*., 2022). Previous studies that have found a response of fern stomata to exogenous ABA applied to epidermal peels (Cai *et al*., 2017) or by spraying onto leaves in plants under specific growth conditions (Hõrak *et al*., 2017; Plackett *et al*., 2021), have found the magnitude of response to be substantially smaller than that of angiosperms, indicative of a difference in ABA sensitivity between ferns and angiosperms.

We further investigated the molecular mechanism underlying this difference between fern and angiosperm stomatal ABA responses. In angiosperms, orthologs of the anion channels SLAC1 and SLAH3 play a key role in stomatal closure responses to ABA, after activation by OST1‐, CPK‐ or CIPK‐type kinases (see Hedrich & Geiger, 2017). We previously found that two *C. richardii* SLAC/SLAH family members – CrSLAC1a and CrSLAC1b – were not activated by Arabidopsis kinase AtOST1 or the fern OST1‐subclade kinases GAMETOPHYTES ABA INSENSITIVE ON A_CE_1 (CrGAIA1) and PARALOG OF GAIA1 (CrPGAI) using the *Xenopus* oocyte expression system (McAdam *et al*., 2016a). In this study, we identified four additional SLAC/SLAH family members (CrSLAC1c‐f) expressed in *C. richardii* sporophytes (Fig. 2) and/or gametophytes (Atallah *et al*., 2018). *CrSLAC1a*‐*c* are expressed at higher levels in guard cells than whole leaf samples, as are two SLAC/SLAH family members in *P. vulgare* (*PvSLAC1a* and *PvSLAC1b*; Figs 2, S3, S4).

Using the *Xenopus* oocyte expression system together with the double‐electrode voltage‐clamp technique, we tested whether any of the newly isolated fern *C. richardii* and *P. vulgare* SLAC homologues are activated by kinases that activate AtSLAC1 from the angiosperm Arabidopsis. Specifically, we tested Arabidopsis ABA‐signalling kinases, all guard cell‐expressed kinases from the SnRK2 OST1‐subclade in the two fern species, and a guard cell‐enriched CPK from *C. richardii* (Figs 3d, S5). Each of these kinases interacted with the fern SLAC homologues (Fig. S6) and induced strong anion channel currents when co‐expressed with AtSLAC1, but did not trigger increased currents with any of the *P. vulgare* guard cell‐enriched SLAC homologues, or any of the *C. richardii* SLAC homologues expressed in currently available transcriptomes (Figs 3d, S3).

To confirm that the fern SLAC genes encode anion channels, we constructed CrSLAC1a F592A channel mutants, wherein the channel is gated open, similar to AtSLAC1 F450A (Chen *et al*., 2010; J. Zhang *et al*., 2018). We measured strong currents for the CrSLAC1a F592A mutant when co‐expressed with AtOST1, but only very small background currents with CrSLAC1a F592A expressed alone (Fig. S3). This confirms that the fern anion channel can be activated in the absence of gating restrictions.

Overall, these results indicate that fern anion channels from the SLAC/SLAH family are not activated by the same kinases as angiosperm SLAC1 and SLAH3 orthologs that facilitate ABA‐mediated stomatal closure. This finding is consistent with the lack of stomatal response to ABA we measured in *P. vulgare*. These results suggest that mechanisms for activation of these anion channels by ABA‐dependent signalling kinases either (1) evolved after divergence of ferns from a common ancestor with seed plants, or (2) evolved earlier and were lost in ferns.

### Moss and hornwort SLAC homologues can be activated by OST1

**Fig. 4:**
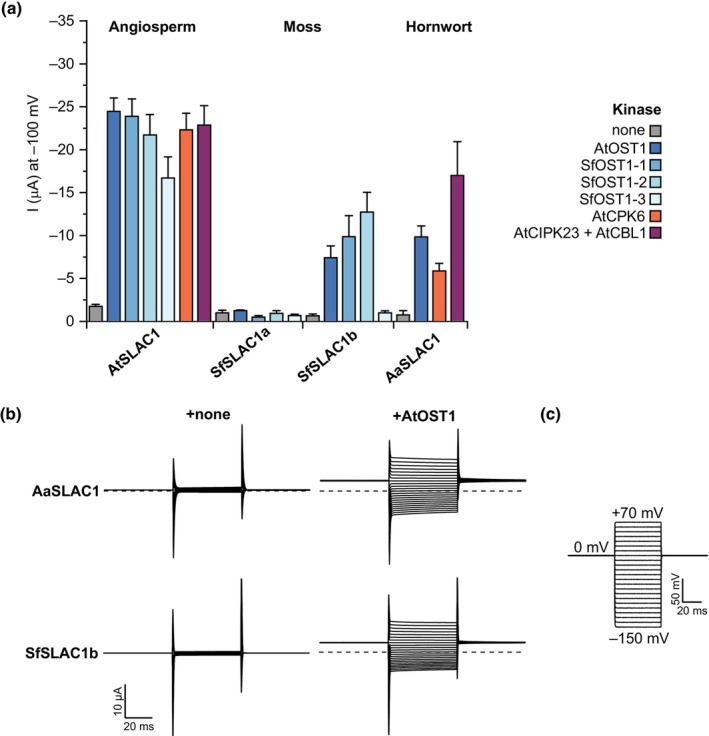
Moss and hornwort SLOW ANION CHANNEL (SLAC) homologues can be activated by abscisic acid (ABA)‐signalling kinases. (a) Mean whole‐oocyte current measurements at −100 mV in nitrate‐based solution with SLAC homologues from the angiosperm *Arabidopsis thaliana*, the moss *Sphagnum fallax* and the hornwort *Anthoceros agrestis*, co‐expressed with or without the indicated kinases in *Xenopus* oocytes (mean ± SEM, *n* ≥ 4). (b) Representative whole‐oocyte currents of SLAC homologues from *A. agrestis* and *S. fallax* expressed either alone or with AtOST1, recorded in nitrate‐based solution. (c) Voltage pulse protocol used for current measurements.

Given the previously published finding of an active SLAC1‐OST1 pair from the moss *P. patens* (PpSLAC1‐PpOST1.2; Lind *et al*., 2015), we further examined the evolution of SLAC activity using the distantly related moss *Sphagnum fallax* and the hornwort *Anthoceros agrestis* to determine whether other bryophytes share kinase‐activated SLAC homologues. There has long been doubt over the phylogenetic relationship between hornworts and other land plants, but numerous recent studies have supported a monophyletic relationship between bryophytes (e.g. Leebens‐Mack *et al*., 2019; Harris *et al*., 2020, 2022; Su *et al*., 2021). We searched available genome sequences and identified the presence of a single SLAC/SLAH family member in *A. agrestis*, and two orthologs of *PpSLAC1* and three members of the OST1 subclade of SnRK2s in *S. fallax* (Table S4; Figs S4, S5), and were able to isolate all of these genes from gametophyte tissues. We found that both *S. fallax* and *A. agrestis* each possess a SLAC homologue that can be activated by ABA‐signalling kinases in the *Xenopus* system (Fig. 4), similar to *P. patens* (Lind *et al*., 2015). This indicates that among bryophytes, moss and hornwort species possess kinase‐activated SLAC homologues, consistent with either an early origin for this mechanism, or independent gain/s in bryophytes.

### Activation in gymnosperm and ‘basal angiosperm’ lineages requires auxiliary factors

**Fig. 5:**
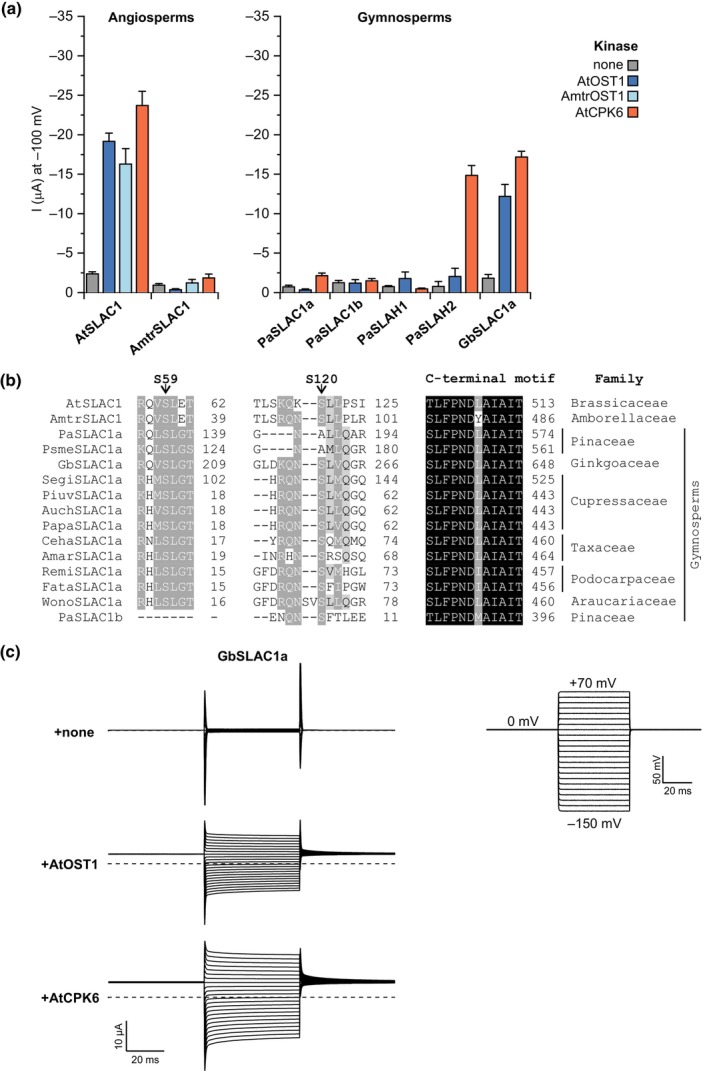
Seed plants differ in terms of SLOW ANION CHANNEL 1 (SLAC1) activity, but SLAC1 HOMOLOGUE 2/3 (SLAH2/3) channel activity may be sufficient for sensitivity to abscisic acid (ABA)‐signalling kinases. (a) Mean whole‐oocyte current measurements at −100 mV in nitrate‐based solution with SLAC/SLAH channels from the angiosperms *Arabidopsis thaliana* and *Amborella trichopoda*, and the gymnosperms *Picea abies* and the *Ginkgo biloba*, co‐expressed with or without kinases in *Xenopus* oocytes (mean ± SEM, *n* ≥ 4). (b) Alignment of protein regions surrounding AtSLAC1 residues S59, S120 and the C‐terminal motif important for activation by ABA‐signalling kinases, showing S59 is not conserved in PaSLAC1b, S120 is conserved in seed plants except members of the gymnosperm Pinaceae family, and the C‐terminal motif is not conserved in AmtrSLAC1. Shading levels indicate the degree of conservation (black = 100%, dark grey ≥ 80% and light grey ≥ 60%). Numbers indicate the position in each protein sequence. Family is indicated for each species. Full sequence details are given in Supporting Information Table S4. (c) Example whole‐oocyte currents of GbSLAC1a, either expressed alone (upper left), with AtOST1 (middle left) or with AtCPK6 (bottom left) recorded in nitrate‐based solution using the indicated voltage pulse protocol (right).

Seed plants (gymnosperms and angiosperms) are the only plants to possess separate orthologs of Arabidopsis SLAC1, SLAH2/3 and SLAH1/4 that are strongly supported by phylogenetic analyses (Fig. S4; Dreyer *et al*., 2012; Sussmilch *et al*., 2019a). So far, the only seed plant SLAC1 channels to be examined have been from the core angiosperm lineages – dicots and monocots; SLAC1 activation has not previously been studied in gymnosperms and ‘basal angiosperm’ lineages. To further examine the evolution of SLAC1 activity in seed plants, we made use of the model gymnosperm *Picea abies*, which shows stomatal sensitivity to ABA, similar to angiosperms (Mayr *et al*., 2012). The *P. abies* genome encodes four SLAC/SLAH proteins – PaSLAC1a, PaSLAC1b (co‐orthologs of AtSLAC1), PaSLAH1 (an ortholog of AtSLAH1 and AtSLAH4) and PaSLAH2 (an ortholog of AtSLAH2 and AtSLAH3; Fig. S4; Table S4). Surprisingly, the *P. abies* SLAC1 co‐orthologs failed to show S‐type anion channel currents when co‐expressed with OST1 in *Xenopus* oocytes (Fig. 5a).

We examined the protein sequences for amino acid differences that could account for the difference in activity between angiosperm and *P. abies* SLAC1 co‐orthologs. We found that serine and threonine residues that are required (although not sufficient) for activation of SLAC1 proteins by OST1 and CPKs (Geiger *et al*., 2009; Brandt *et al*., 2015; Deng *et al*., 2021) are missing in PaSLAC1a in the region corresponding to AtSLAC1 S120, and in PaSLAC1b in the region of AtSLAC1 S59 (Fig. 5b). Further examination showed that SLAC1 ortholog sequences of other species from the gymnosperm Pinaceae family similarly lack S120 (Fig. 5b). However, in species from other gymnosperm families, including *Ginkgo biloba*, a serine is present at these important positions. We tested the activity of this *G. biloba* SLAC1 (GbSLAC1a) and found that it could be activated by OST1 and CPKs (Fig. 5a,c). In contrast to the *P. abies* SLAC1 co‐orthologs, WT PaSLAH2 (an ortholog of AtSLAH2 and AtSLAH3) yielded substantial currents in nitrate‐based media in the presence of a CPK (Fig. 5a), similar to AtSLAH3 (Geiger *et al*., 2011). These results indicate that mechanisms for kinase activation of SLAC1 channels are present in some gymnosperms but have been lost from the Pinaceae family, where ABA‐signalling kinases activate SLAH2/3 orthologs only.

To further examine the evolution of SLAC1 activity in seed plants, we made use of the single living representative of the Amborellales – the sister lineage to all other extant flowering plants – *Amborella trichopoda* (Albert *et al*., 2013). The *A. trichopoda* genome encodes a single SLAC1 ortholog (AmtrSLAC1), two orthologs of SLAH2/3 and a single ortholog of AtOST1/AtSnRK2.2/AtSnRK2.3 (AmtrOST1; Figs S4, S5; Table S4). We examined the activity of AmtrSLAC1 when co‐expressed with AtOST1, AmtrOST1 or AtCPK6, and found that it was insensitive to activation from any of these ABA‐signalling kinases (Fig. 5a).

## Discussion

We have found that guard cells of the fern models *C. richardii* and *P. vulgare* are equipped with ABA biosynthesis and signalling pathways (Figs 2, S2). Previous studies have suggested that the expression of ABA signalling and ion channel homologues in stomata‐bearing tissues indicates a potential conserved role in ABA‐mediated stomatal closure (e.g. Cai *et al*., 2017; Plackett *et al*., 2021). However, we have found that ABA‐signalling kinases are unable to activate guard cell SLAC homologues from the ferns *P. vulgare* and *C. richardii* (Fig. 3). These results are consistent with our own results showing *P. vulgare* stomata to be insensitive to ABA application (Fig. 3), and previous findings of a lack of stomatal closure response to endogenous ABA levels in ferns (Brodribb & McAdam, 2011; McAdam & Brodribb, 2012; Cândido‐Sobrinho *et al*., 2022).

Furthermore, these results account for the lack of stomatal phenotype for ABA‐insensitive fern mutants (McAdam *et al*., 2016a). These fern mutants show sharp contrast to the wilty phenotypes characteristic of ABA biosynthesis and signalling mutants in angiosperm species with rapid stomatal closure in response to increases in endogenous ABA levels (Merlot *et al*., 2002; Mustilli *et al*., 2002; Merilo *et al*., 2013; McAdam *et al*., 2015). Further emphasising differences between the stomatal responses of seed plants and other vascular plant groups, there has been found to be a lack of ABA‐mediated K^+^ efflux from the guard cells of fern and lycophyte species (Gong *et al*., 2021). Together, these results suggest that SLAC‐like channels are not required for drought‐induced stomatal closure in nonflowering plants, but instead may be involved in nutrient movement, similar to SLAH channels in angiosperms (Maierhofer *et al*., 2014b; Cubero‐Font *et al*., 2016; Qiu *et al*., 2016).

Ferns synthesise ABA in response to dehydration stress, similar to all other land plant groups (e.g. Hartung *et al*., 1987; Hellwege *et al*., 1994; Qin & Zeevaart, 1999; McAdam & Brodribb, 2012; Xiao *et al*., 2018; Xu *et al*., 2018), consistent with an ancient role for ABA in desiccation tolerance (e.g. Khandelwal *et al*., 2010; Tougane *et al*., 2010; Komatsu *et al*., 2013; Jahan *et al*., 2019; Shinozawa *et al*., 2019). In response to slow dehydration, ABA triggers the upregulation of proteins that function in osmoregulation to protect fragile cellular components during desiccation, including metabolic enzymes, sugar transporters and aquaporins, in diverse plant species including bryophytes, lycophytes, ferns and angiosperms (e.g. Reynolds & Bewley, 1993; Hellwege *et al*., 1994; Cuming *et al*., 2007; Wang *et al*., 2010; VanBuren *et al*., 2017; Jahan *et al*., 2019; Shinozawa *et al*., 2019). In line with this function, ABA does induce changes in gene expression in ferns including in guard cells (Plackett *et al*., 2021). Thus, although our results indicate that ABA signalling is not involved in rapid stomatal closure via ion channel activation as occurs in seed plants, it is likely that ABA signalling is involved in slow dehydration tolerance mechanisms in ferns, similar to other land plant lineages.

We find kinase‐sensitive SLAC homologues in the moss *S. fallax* and the hornwort *A. agrestis* (Figs 3, 4, 5), where they might serve plant anion transport functions. It is not physically possible for SLAC homologues to play any role in stomatal closure in *S. fallax* and *A. agrestis*, as stomata ‘open’ by irreversible guard cell collapse in line with a predominant role for moss and hornwort stomata in promoting desiccation for spore drying and dispersal in these species (Duckett *et al*., 2009; Merced, 2015; Renzaglia *et al*., 2017). In line with an ancient role for ABA in vegetative desiccation tolerance (see McAdam & Sussmilch, 2021), application of exogenous ABA improves desiccation survival in *S. fallax* (Nibau *et al*., 2022). Although future mutant studies will be required to determine the precise roles of SLAC homologues in bryophytes, it is possible that a role for SLAC homologues in nutrient movement – similar to SLAH channels in angiosperms (Maierhofer *et al*., 2014b; Cubero‐Font *et al*., 2016; Qiu *et al*., 2016) – may have been co‐opted for ABA‐dependent ion movement associated with desiccation tolerance in gametophyte tissues in mosses and hornworts.

We have found sensitivity to ABA‐signalling kinases to be lacking for SLAC homologues from the alga *K. nitens*, liverwort *M. polymorpha* and lycophyte *S. moellendorffii*, in addition to the ferns *C. richardii* and *P. vulgare* and SLAC1 orthologs of seed plants *P. abies* and *A. trichopoda* (Figs 3, 4, 5; Lind *et al*., 2015; McAdam *et al*., 2016a). The lack of activation of the seed plant SLAC1 orthologs was particularly unexpected, given the shared ABA‐mediated stomatal closure response in these angiosperm and gymnosperm species (Mayr *et al*., 2012; McAdam & Brodribb, 2015). However, it is possible that activation of SLAH2/3 orthologs by ABA‐signalling kinases is sufficient for this response in these species.

**Fig. 6:**
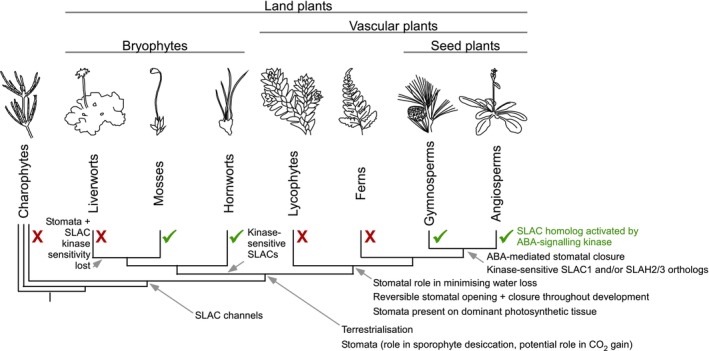
Model for the timing of key events during stomatal evolution. The hypothesised timing of key traits is indicated with grey arrows on the current model of land plant phylogeny (Leebens‐Mack *et al*., 2019; Harris *et al*., 2020). The presence or absence of evidence for SLOW ANION CHANNEL (SLAC) activation by abscisic acid (ABA)‐signalling kinases for each major plant group is indicated with a tick or cross, respectively, based on the combined results of this study and previous findings (Geiger *et al*., 2009; Lind *et al*., 2015; McAdam *et al*., 2016a). Branch lengths are not to scale.

Overall, we find evidence of a complex evolutionary history for SLAC sensitivity to ABA‐signalling kinases with either (1) multiple gains or (2) an early gain for a nonguard cell‐specific functional origin and subsequent losses (Fig. 6). Given the lack of this trait in lycophytes and ferns, we propose that bryophytes and seed plants may have separately co‐opted SLAC/SLAH channels for different roles downstream of the ABA‐signalling pathway, with seed plants using ABA to trigger rapid stomatal closure and some bryophytes using ABA for osmoregulation more generally in other cell types, including vegetative tissues that lack stomata. Our results explain differences in ABA sensitivity between some seed plants and ferns, while giving new insight into the transcriptional features of fern guard cells. These findings highlight the importance of studies directly testing if the roles of genes and the signal‐dependent activation of encoded proteins are conserved in different plant species, in order to understand the evolution of plant signalling processes.

## Competing interests

None declared.

## Author contributions

FCS, TM, JH, LJV, CL, MM, HMM, MSB and JS performed the research and analysed the data. FCS, PA, KFXM, DB, MRGR, DG, JS and RH designed the study. FCS, MRGR, DG, JS and RH wrote the manuscript.

## Supporting information


**Fig. S1** Most common annotated domains of differentially expressed orthogroups.
**Fig. S2** Relative expression of orthogroups containing abscisic acid biosynthesis pathway components.
**Fig. S3** Additional data for fern SLAC homologue activity and expression.
**Fig. S4** Phylogeny of the SLAC/SLAH family in streptophytes.
**Fig. S5** Phylogeny of the SnRK2 family in streptophytes.
**Fig. S6** Bimolecular fluorescence complementation experiments showing interaction between fern SLAC homologues and kinases tested in oocytes.
**Table S1** Overview of transcriptomes and differential gene expression analysis.
**Table S2** Genomic data used for gene expression analysis and evolutionary reconstruction.

**Table S3** Complete list of differentially expressed orthogroups from Fig. 1.

**Table S4** Gene sequence details.
**Table S5** Primer details.

**Table S6** Orthogroup details for genes of interest for Fig. 2.
Please note: Wiley is not responsible for the content or functionality of any Supporting Information supplied by the authors. Any queries (other than missing material) should be directed to the *New Phytologist* Central Office.

## Data Availability

Arabidopsis and fern sequences are accessible under the GenBank project accessions PRJNA731641 and PRJEB45027. Barley sequences are accessible at EMBL‐EBI ArrayExpress under E‐MTAB‐10534. Orthogroup sequence details for genes of interest are listed in Table S6. All other gene accessions are listed in Table S4.

## References

[nph20172-bib-0001] Albert VA , Barbazuk WB , dePamphilis CW , Der JP , Leebens‐Mack J , Ma H , Palmer JD , Rounsley S , Sankoff D , Schuster SC *et al*. 2013. The *Amborella* genome and the evolution of flowering plants. Science 342: 1241089. 24357323 10.1126/science.1241089

[nph20172-bib-0002] Atallah NM , Vitek O , Gaiti F , Tanurdzic M , Banks JA . 2018. Sex determination in *Ceratopteris richardii* is accompanied by transcriptome changes that drive epigenetic reprogramming of the young gametophyte. G3: Genes, Genomes, Genetics 8: 2205–2214. 29720393 10.1534/g3.118.200292 PMC6027899

[nph20172-bib-0003] Bauer H , Ache P , Lautner S , Fromm J , Hartung W , Al‐Rasheid KAS , Sonnewald S , Sonnewald U , Kneitz S , Lachmann N *et al*. 2013. The stomatal response to reduced relative humidity requires guard cell‐autonomous ABA synthesis. Current Biology 23: 53–57. 23219726 10.1016/j.cub.2012.11.022

[nph20172-bib-0004] Becker D , Dreyer I , Hoth S , Reid JD , Busch H , Lehnen M , Palme K , Hedrich R . 1996. Changes in voltage activation, Cs^+^ sensitivity, and ion permeability in H5 mutants of the plant K^+^ channel KAT1. Proceedings of the National Academy of Sciences, USA 93: 8123–8128. 10.1073/pnas.93.15.8123 PMC38886 8755614

[nph20172-bib-0005] Bienert MD , Diehn TA , Richet N , Chaumont F , Bienert GP . 2018. Heterotetramerization of plant PIP1 and PIP2 aquaporins is an evolutionary ancient feature to guide PIP1 plasma membrane localization and function. Frontiers in Plant Science 9: 382. 29632543 10.3389/fpls.2018.00382 PMC5879115

[nph20172-bib-0006] Blum M , Chang H‐Y , Chuguransky S , Grego T , Kandasaamy S , Mitchell A , Nuka G , Paysan‐Lafosse T , Qureshi M , Raj S *et al*. 2021. The InterPro protein families and domains database: 20 years on. Nucleic Acids Research 49(D1): D344–D354. 33156333 10.1093/nar/gkaa977 PMC7778928

[nph20172-bib-0007] Bowles AMC , Paps J , Bechtold U . 2022. Water‐related innovations in land plants evolved by different patterns of gene cooption and novelty. New Phytologist 235: 732–742. 35048381 10.1111/nph.17981 PMC9303528

[nph20172-bib-0008] Bowman JL , Kohchi T , Yamato KT , Jenkins J , Shu S , Ishizaki K , Yamaoka S , Nishihama R , Nakamura Y , Berger F *et al*. 2017. Insights into land plant evolution garnered from the *Marchantia polymorpha* genome. Cell 171: 287–304. 28985561 10.1016/j.cell.2017.09.030

[nph20172-bib-0009] Brandt B , Munemasa S , Wang C , Nguyen D , Yong T , Yang PG , Poretsky E , Belknap TF , Waadt R , Alemán F *et al*. 2015. Calcium specificity signaling mechanisms in abscisic acid signal transduction in Arabidopsis guard cells. eLife 4: e03599. 26192964 10.7554/eLife.03599 PMC4507714

[nph20172-bib-0010] Brodribb TJ , McAdam SAM . 2011. Passive origins of stomatal control in vascular plants. Science 331: 582–585. 21163966 10.1126/science.1197985

[nph20172-bib-0011] Cai S , Chen G , Wang Y , Huang Y , Marchant B , Yang Q , Dai F , Hills A , Franks PJ , Nevo E *et al*. 2017. Evolutionary conservation of ABA signaling for stomatal closure. Plant Physiology 174: 732–747. 28232585 10.1104/pp.16.01848 PMC5462018

[nph20172-bib-0012] Cândido‐Sobrinho SA , Lima VF , Freire FBS , de Souza LP , Gago J , Fernie AR , Daloso DM . 2022. Metabolism‐mediated mechanisms underpin the differential stomatal speediness regulation among ferns and angiosperms. Plant, Cell & Environment 45: 296–311. 10.1111/pce.14232 34800300

[nph20172-bib-0013] Chater C , Caine RS , Tomek M , Wallace S , Kamisugi Y , Cuming AC , Lang D , MacAlister CA , Casson S , Bergmann DC *et al*. 2016. Origin and function of stomata in the moss *Physcomitrella patens* . Nature Plants 2: 16179. 27892923 10.1038/nplants.2016.179 PMC5131878

[nph20172-bib-0014] Chater C , Kamisugi Y , Movahedi M , Fleming A , Cuming AC , Gray JE , Beerling DJ . 2011. Regulatory mechanism controlling stomatal behavior conserved across 400 million years of land plant evolution. Current Biology 21: 1025–1029. 21658944 10.1016/j.cub.2011.04.032

[nph20172-bib-0015] Chater CCC . 2024. A tail of two horses? Guard cell abscisic acid and carbon dioxide signalling in the Equisetum ferns. New Phytologist 243: 503–505. 38453694 10.1111/nph.19659

[nph20172-bib-0016] Chen Y‐h , Hu L , Punta M , Bruni R , Hillerich B , Kloss B , Rost B , Love J , Siegelbaum SA , Hendrickson WA . 2010. Homologue structure of the SLAC1 anion channel for closing stomata in leaves. Nature 467: 1074–1080. 20981093 10.1038/nature09487 PMC3548404

[nph20172-bib-0017] Cheng W‐H , Endo A , Zhou L , Penney J , Chen H‐C , Arroyo A , Leon P , Nambara E , Asami T , Seo M *et al*. 2002. A unique short‐chain dehydrogenase/reductase in Arabidopsis glucose signaling and abscisic acid biosynthesis and functions. Plant Cell 14: 2723–2743. 12417697 10.1105/tpc.006494 PMC152723

[nph20172-bib-0018] Clark JW , Harris BJ , Hetherington AJ , Hurtado‐Castano N , Brench RA , Casson S , Williams TA , Gray JE , Hetherington AM . 2022. The origin and evolution of stomata. Current Biology 32: R539–R553. 35671732 10.1016/j.cub.2022.04.040

[nph20172-bib-0019] Cubero‐Font P , Maierhofer T , Jaslan J , Rosales Miguel A , Espartero J , Díaz‐Rueda P , Müller Heike M , Hürter A‐L , Al‐Rasheid Khaled AS , Marten I *et al*. 2016. Silent S‐type anion channel subunit SLAH1 gates SLAH3 open for chloride root‐to‐shoot translocation. Current Biology 26: 2213–2220. 27397895 10.1016/j.cub.2016.06.045

[nph20172-bib-0020] Cuming AC , Cho SH , Kamisugi Y , Graham H , Quatrano RS . 2007. Microarray analysis of transcriptional responses to abscisic acid and osmotic, salt, and drought stress in the moss, *Physcomitrella patens* . New Phytologist 176: 275–287. 17696978 10.1111/j.1469-8137.2007.02187.x

[nph20172-bib-0021] Dadacz‐Narloch B , Beyhl D , Larisch C , López‐Sanjurjo EJ , Reski R , Kuchitsu K , Müller TD , Becker D , Schönknecht G , Hedrich R . 2011. A novel calcium binding site in the slow vacuolar cation channel TPC1 senses luminal calcium levels. Plant Cell 23: 2696–2707. 21764990 10.1105/tpc.111.086751 PMC3226227

[nph20172-bib-0022] Deng Y‐n , Kashtoh H , Wang Q , Zhen G‐x , Li Q‐y , Tang L‐h , Gao H‐l , Zhang C‐r , Qin L , Su M *et al*. 2021. Structure and activity of SLAC1 channels for stomatal signaling in leaves. Proceedings of the National Academy of Sciences, USA 118: e2015151118. 10.1073/pnas.2015151118 PMC8106318 33926963

[nph20172-bib-0023] Dreyer I , Gomez‐Porras JL , Riaño‐Pachón DM , Hedrich R , Geiger D . 2012. Molecular evolution of slow and quick anion channels (SLACs and QUACs/ALMTs). Frontiers in Plant Science 3: 263. 23226151 10.3389/fpls.2012.00263 PMC3509319

[nph20172-bib-0024] Duckett JG , Pressel S . 2018. The evolution of the stomatal apparatus: intercellular spaces and sporophyte water relations in bryophytes – two ignored dimensions. Philosophical Transactions of the Royal Society of London B: Biological Sciences 373: 20160498. 29254963 10.1098/rstb.2016.0498 PMC5745334

[nph20172-bib-0025] Duckett JG , Pressel S . 2022. Do moss sporophytes maintain water balance? New insights from sporophyte water relations and the wild maturation cycle in *Funaria hygrometrica* Hedw. Journal of Bryology 44: 187–198.

[nph20172-bib-0026] Duckett JG , Pressel S , P'ng KMY , Renzaglia KS . 2009. Exploding a myth: the capsule dehiscence mechanism and the function of pseudostomata in *Sphagnum* . New Phytologist 183: 1053–1063. 19552695 10.1111/j.1469-8137.2009.02905.x

[nph20172-bib-0027] Emms DM , Kelly S . 2015. orthofinder: solving fundamental biases in whole genome comparisons dramatically improves orthogroup inference accuracy. Genome Biology 16: 157. 26243257 10.1186/s13059-015-0721-2 PMC4531804

[nph20172-bib-0028] Fontaine J‐X , Tercé‐Laforgue T , Armengaud P , Clément G , Renou J‐P , Pelletier S , Catterou M , Azzopardi M , Gibon Y , Lea PJ *et al*. 2012. Characterization of a NADH‐dependent glutamate dehydrogenase mutant of Arabidopsis demonstrates the key role of this enzyme in root carbon and nitrogen metabolism. Plant Cell 24: 4044–4065. 23054470 10.1105/tpc.112.103689 PMC3517235

[nph20172-bib-0029] Geiger D , Maierhofer T , AL‐Rasheid KA , Scherzer S , Mumm P , Liese A , Ache P , Wellmann C , Marten I , Grill E . 2011. Stomatal closure by fast abscisic acid signaling is mediated by the guard cell anion channel SLAH3 and the receptor RCAR1. Science Signaling 4: ra32. 21586729 10.1126/scisignal.2001346

[nph20172-bib-0030] Geiger D , Scherzer S , Mumm P , Marten I , Ache P , Matschi S , Liese A , Wellmann C , Al‐Rasheid KAS , Grill E *et al*. 2010. Guard cell anion channel SLAC1 is regulated by CDPK protein kinases with distinct Ca^2+^ affinities. Proceedings of the National Academy of Sciences, USA 107: 8023–8028. 10.1073/pnas.0912030107 PMC2867891 20385816

[nph20172-bib-0031] Geiger D , Scherzer S , Mumm P , Stange A , Marten I , Bauer H , Ache P , Matschi S , Liese A , Al‐Rasheid KA *et al*. 2009. Activity of guard cell anion channel SLAC1 is controlled by drought‐stress signaling kinase‐phosphatase pair. Proceedings of the National Academy of Sciences, USA 106: 21425–21430. 10.1073/pnas.0912021106 PMC2795561 19955405

[nph20172-bib-0032] Gong L , Liu X‐D , Zeng Y‐Y , Tian X‐Q , Li Y‐L , Turner NC , Fang X‐W . 2021. Stomatal morphology and physiology explain varied sensitivity to abscisic acid across vascular plant lineages. Plant Physiology 186: 782–797. 33620497 10.1093/plphys/kiab090 PMC8154066

[nph20172-bib-0033] González‐Guzmán M , Apostolova N , Bellés JM , Barrero JM , Piqueras P , Ponce MR , Micol JL , Serrano R , Rodríguez PL . 2002. The short‐chain alcohol dehydrogenase ABA2 catalyzes the conversion of xanthoxin to abscisic aldehyde. Plant Cell 14: 1833–1846. 12172025 10.1105/tpc.002477 PMC151468

[nph20172-bib-0034] Grabherr MG , Haas BJ , Yassour M , Levin JZ , Thompson DA , Amit I , Adiconis X , Fan L , Raychowdhury R , Zeng Q . 2011. Full‐length transcriptome assembly from RNA‐Seq data without a reference genome. Nature Biotechnology 29: 644–652. 10.1038/nbt.1883 PMC3571712 21572440

[nph20172-bib-0035] Guindon S , Dufayard JF , Lefort V , Anisimova M , Hordijk W , Gascuel O . 2010. New algorithms and methods to estimate maximum‐likelihood phylogenies: assessing the performance of phyml 3.0. Systematic Biology 59: 307–321. 20525638 10.1093/sysbio/syq010

[nph20172-bib-0036] Harris BJ , Clark JW , Schrempf D , Szöllősi GJ , Donoghue PCJ , Hetherington AM , Williams TA . 2022. Divergent evolutionary trajectories of bryophytes and tracheophytes from a complex common ancestor of land plants. Nature Ecology & Evolution 6: 1634–1643. 36175544 10.1038/s41559-022-01885-x PMC9630106

[nph20172-bib-0037] Harris BJ , Harrison CJ , Hetherington AM , Williams TA . 2020. Phylogenomic evidence for the monophyly of bryophytes and the reductive evolution of stomata. Current Biology 30: 2001–2012. 32302587 10.1016/j.cub.2020.03.048

[nph20172-bib-0038] Hartung W , Weiler EW , Volk OH . 1987. Immunochemical evidence that abscisic acid is produced by several species of Anthocerotae and Marchantiales. Bryologist 90: 393–400.

[nph20172-bib-0039] Hedrich R , Geiger D . 2017. Biology of SLAC1‐type anion channels – from nutrient uptake to stomatal closure. New Phytologist 216: 46–61. 28722226 10.1111/nph.14685

[nph20172-bib-0040] Hellwege EM , Dietz K‐J , Volk OH , Hartung W . 1994. Abscisic acid and the induction of desiccation tolerance in the extremely xerophilic liverwort *Exormotheca holstii* . Planta 194: 525–531.

[nph20172-bib-0041] Hickok LG , Warne TR , Fribourg RS . 1995. The biology of the fern Ceratopteris and its use as a model system. International Journal of Plant Sciences 156: 332–345.

[nph20172-bib-0042] Hickok LG , Warne TR , Slocum MK . 1987. *Ceratopteris richardii*: applications for experimental plant biology. American Journal of Botany 74: 1304–1316.

[nph20172-bib-0043] Hõrak H , Kollist H , Merilo E . 2017. Fern stomatal responses to ABA and CO_2_ depend on species and growth conditions. Plant Physiology 174: 672–679. 28351911 10.1104/pp.17.00120 PMC5462029

[nph20172-bib-0044] Huang S , Maierhofer T , Hashimoto K , Xu X , Karimi SM , Müller H , Geringer MA , Wang Y , Kudla J , De Smet I *et al*. 2023. The CIPK23 protein kinase represses SLAC1‐type anion channels in Arabidopsis guard cells and stimulates stomatal opening. New Phytologist 238: 270–282. 36597715 10.1111/nph.18708

[nph20172-bib-0045] Huang S , Waadt R , Nuhkat M , Kollist H , Hedrich R , Roelfsema MRG . 2019. Calcium signals in guard cells enhance the efficiency by which abscisic acid triggers stomatal closure. New Phytologist 224: 177–187. 31179540 10.1111/nph.15985 PMC6771588

[nph20172-bib-0046] Jahan A , Komatsu K , Wakida‐Sekiya M , Hiraide M , Tanaka K , Ohtake R , Umezawa T , Toriyama T , Shinozawa A , Yotsui I *et al*. 2019. Archetypal roles of an abscisic acid receptor in drought and sugar responses in liverworts. Plant Physiology 179: 317–328. 30442644 10.1104/pp.18.00761 PMC6324230

[nph20172-bib-0047] Khandelwal A , Cho SH , Marella H , Sakata Y , Perroud P‐F , Pan A , Quatrano RS . 2010. Role of ABA and ABI3 in desiccation tolerance. Science 327: 546. 20110497 10.1126/science.1183672

[nph20172-bib-0048] Komatsu K , Suzuki N , Kuwamura M , Nishikawa Y , Nakatani M , Ohtawa H , Takezawa D , Seki M , Tanaka M , Taji T *et al*. 2013. Group A PP2Cs evolved in land plants as key regulators of intrinsic desiccation tolerance. Nature Communications 4: 2219. 10.1038/ncomms3219 PMC3731658 23900426

[nph20172-bib-0049] Lange OL , Lösch R , Schulze ED , Kappen L . 1971. Responses of stomata to changes in humidity. Planta 100: 76–86. 24488104 10.1007/BF00386887

[nph20172-bib-0050] Leebens‐Mack JH , Barker MS , Carpenter EJ , Deyholos MK , Gitzendanner MA , Graham SW , Grosse I , Li Z , Melkonian M , Mirarab S *et al*. 2019. One thousand plant transcriptomes and the phylogenomics of green plants. Nature 574: 679–685. 31645766 10.1038/s41586-019-1693-2 PMC6872490

[nph20172-bib-0051] Lefort V , Longueville JE , Gascuel O . 2017. SMS: smart model selection in PhyML. Molecular Biology and Evolution 34: 2422–2424. 28472384 10.1093/molbev/msx149 PMC5850602

[nph20172-bib-0052] Li B , Dewey CN . 2011. RSEM: accurate transcript quantification from RNA‐Seq data with or without a reference genome. BMC Bioinformatics 12: 323. 21816040 10.1186/1471-2105-12-323 PMC3163565

[nph20172-bib-0053] Li X , Xie Y , Zhang Q , Hua X , Peng L , Li K , Yu Q , Chen Y , Yao H , He J *et al*. 2022. Monomerization of abscisic acid receptors through CARKs‐mediated phosphorylation. New Phytologist 235: 533–549. 35388459 10.1111/nph.18149

[nph20172-bib-0054] Li Y , Ding Y , Qu L , Li X , Lai Q , Zhao P , Gao Y , Xiang C , Cang C , Liu X *et al*. 2022. Structure of the Arabidopsis guard cell anion channel SLAC1 suggests activation mechanism by phosphorylation. Nature Communications 13: 2511. 10.1038/s41467-022-30253-3 PMC9076830 35523967

[nph20172-bib-0055] Lind C , Dreyer I , López‐Sanjurjo EJ , von Meyer K , Ishizaki K , Kohchi T , Lang D , Zhao Y , Kreuzer I , Al‐Rasheid KAS *et al*. 2015. Stomatal guard cells co‐opted an ancient ABA‐dependent desiccation survival system to regulate stomatal closure. Current Biology 25: 928–935. 25802151 10.1016/j.cub.2015.01.067

[nph20172-bib-0056] Lösch R . 1979. Responses of stomata to environmental factors‐experiments with isolated epidermal strips of *Polypodium vulgare* . Oecologia 39: 229–238. 28309439 10.1007/BF00348071

[nph20172-bib-0057] Love MI , Huber W , Anders S . 2014. Moderated estimation of fold change and dispersion for RNA‐seq data with DESeq2. Genome Biology 15: 550. 25516281 10.1186/s13059-014-0550-8 PMC4302049

[nph20172-bib-0058] Maierhofer T , Diekmann M , Offenborn JN , Lind C , Bauer H , Hashimoto K , Al‐Rasheid KAS , Luan S , Kudla J , Geiger D *et al*. 2014a. Site‐ and kinase‐specific phosphorylation‐mediated activation of SLAC1, a guard cell anion channel stimulated by abscisic acid. Science Signaling 7: ra86. 25205850 10.1126/scisignal.2005703

[nph20172-bib-0059] Maierhofer T , Lind C , Hüttl S , Scherzer S , Papenfuß M , Simon J , Al‐Rasheid KAS , Ache P , Rennenberg H , Hedrich R *et al*. 2014b. A single‐pore residue renders the Arabidopsis root anion channel SLAH2 highly nitrate selective. Plant Cell 26: 2554–2567. 24938289 10.1105/tpc.114.125849 PMC4114951

[nph20172-bib-0060] Malinova I , Kunz H‐H , Alseekh S , Herbst K , Fernie AR , Gierth M , Fettke J . 2014. Reduction of the cytosolic phosphoglucomutase in Arabidopsis reveals impact on plant growth, seed and root development, and carbohydrate partitioning. PLoS ONE 9: e112468. 25401493 10.1371/journal.pone.0112468 PMC4234415

[nph20172-bib-0061] Mayr S , Schmid P , Beikircher B . 2012. Plant water relations in alpine winter. In: Lütz C , ed. Plants in alpine regions: cell physiology of adaption and survival strategies. Vienna, Austria: Springer, 153–162.

[nph20172-bib-0062] McAdam SAM , Brodribb TJ , Banks JA , Hedrich R , Atallah NM , Cai C , Geringer MA , Lind C , Nichols DS , Stachowski K *et al*. 2016a. Abscisic acid controlled sex before transpiration in vascular plants. Proceedings of the National Academy of Sciences, USA 113: 12862–12867. 10.1073/pnas.1606614113 PMC5111647 27791082

[nph20172-bib-0063] McAdam SAM , Brodribb TJ . 2012. Fern and lycophyte guard cells do not respond to endogenous abscisic acid. Plant Cell 24: 1510–1521. 22517320 10.1105/tpc.112.096404 PMC3398560

[nph20172-bib-0064] McAdam SAM , Brodribb TJ . 2015. The evolution of mechanisms driving the stomatal response to vapor pressure deficit. Plant Physiology 167: 833–843. 25637454 10.1104/pp.114.252940 PMC4348763

[nph20172-bib-0065] McAdam SAM , Duckett JG , Sussmilch FC , Pressel S , Renzaglia KS , Hedrich R , Brodribb TJ , Merced A . 2021. Stomata: the holey grail of plant evolution. American Journal of Botany 108: 366–371. 33687736 10.1002/ajb2.1619 PMC8175006

[nph20172-bib-0066] McAdam SAM , Sussmilch FC . 2021. The evolving role of abscisic acid in cell function and plant development over geological time. Seminars in Cell & Developmental Biology 109: 39–45. 32571626 10.1016/j.semcdb.2020.06.006

[nph20172-bib-0067] McAdam SAM , Sussmilch FC , Brodribb TJ . 2016b. Stomatal responses to vapour pressure deficit are regulated by high speed gene expression in angiosperms. Plant, Cell & Environment 39: 485–491. 10.1111/pce.12633 26353082

[nph20172-bib-0068] McAdam SAM , Sussmilch FC , Brodribb TJ , Ross JJ . 2015. Molecular characterization of a mutation affecting abscisic acid biosynthesis and consequently stomatal responses to humidity in an agriculturally important species. AoB Plants 7: plv091. 26216469 10.1093/aobpla/plv091 PMC4583606

[nph20172-bib-0069] McAinsh MR , Brownlee C , Hetherington AM . 1990. Abscisic acid‐induced elevation of guard cell cytosolic Ca^2+^ precedes stomatal closure. Nature 343: 186–188.

[nph20172-bib-0070] Merced A . 2015. Novel insights on the structure and composition of pseudostomata of *Sphagnum* . American Journal of Botany 102: 329–335. 25784466 10.3732/ajb.1400564

[nph20172-bib-0071] Merced A , Renzaglia KS . 2014. Developmental changes in guard cell wall structure and pectin composition in the moss *Funaria*: implications for function and evolution of stomata. Annals of Botany 114: 1001–1010. 25129633 10.1093/aob/mcu165 PMC4171074

[nph20172-bib-0072] Merilo E , Laanemets K , Hu H , Xue S , Jakobson L , Tulva I , Gonzalez‐Guzman M , Rodriguez PL , Schroeder JI , Broschè M *et al*. 2013. PYR/RCAR receptors contribute to ozone‐, reduced air humidity‐, darkness‐, and CO_2_‐induced stomatal regulation. Plant Physiology 162: 1652–1668. 23703845 10.1104/pp.113.220608 PMC3707544

[nph20172-bib-0073] Merlot S , Mustilli A‐C , Genty B , North H , Lefebvre V , Sotta B , Vavasseur A , Giraudat J . 2002. Use of infrared thermal imaging to isolate Arabidopsis mutants defective in stomatal regulation. The Plant Journal 30: 601–609. 12047634 10.1046/j.1365-313x.2002.01322.x

[nph20172-bib-0074] Mittelheuser CJ , Van Steveninck RFM . 1969. Stomatal closure and inhibition of transpiration induced by (RS)‐abscisic acid. Nature 221: 281–282.

[nph20172-bib-0075] Mori IC , Murata Y , Yang Y , Munemasa S , Wang Y‐F , Andreoli S , Tiriac H , Alonso JM , Harper JF , Ecker JR *et al*. 2006. CDPKs CPK6 and CPK3 function in ABA regulation of guard cell S‐type anion‐ and Ca^2+^‐permeable channels and stomatal closure. PLoS Biology 4: e327. 17032064 10.1371/journal.pbio.0040327 PMC1592316

[nph20172-bib-0076] Morris JL , Puttick MN , Clark JW , Edwards D , Kenrick P , Pressel S , Wellman CH , Yang Z , Schneider H , Donoghue PCJ . 2018. The timescale of early land plant evolution. Proceedings of the National Academy of Sciences, USA 115: E2274–E2283. 10.1073/pnas.1719588115 PMC5877938 29463716

[nph20172-bib-0077] Moummou H , Kallberg Y , Tonfack LB , Persson B , van der Rest B . 2012. The plant Short‐Chain Dehydrogenase (SDR) superfamily: genome‐wide inventory and diversification patterns. BMC Plant Biology 12: 1–17. 23167570 10.1186/1471-2229-12-219 PMC3541173

[nph20172-bib-0078] Müller HM , Schäfer N , Bauer H , Geiger D , Lautner S , Fromm J , Riederer M , Bueno A , Nussbaumer T , Mayer K *et al*. 2017. The desert plant *Phoenix dactylifera* closes stomata via nitrate‐regulated SLAC1 anion channel. New Phytologist 216: 150–162. 28670699 10.1111/nph.14672

[nph20172-bib-0079] Mustilli A‐C , Merlot S , Vavasseur A , Fenzi F , Giraudat J . 2002. Arabidopsis OST1 protein kinase mediates the regulation of stomatal aperture by abscisic acid and acts upstream of reactive oxygen species production. Plant Cell 14: 3089–3099. 12468729 10.1105/tpc.007906 PMC151204

[nph20172-bib-0080] Nibau C , van de Koot W , Spiliotis D , Williams K , Kramaric T , Beckmann M , Mur L , Hiwatashi Y , Doonan JH . 2022. Molecular and physiological responses to desiccation indicate the ABA pathway is conserved in the peat moss, Sphagnum. Journal of Experimental Botany 73: 4576–4591. 35383351 10.1093/jxb/erac133 PMC9291362

[nph20172-bib-0081] Nour‐Eldin HH , Hansen BG , Nørholm MHH , Jensen JK , Halkier BA . 2006. Advancing uracil‐excision based cloning towards an ideal technique for cloning PCR fragments. Nucleic Acids Research 34: e122. 17000637 10.1093/nar/gkl635 PMC1635280

[nph20172-bib-0082] Ohashi‐Ito K , Bergmann DC . 2006. Arabidopsis FAMA controls the final proliferation/differentiation switch during stomatal development. Plant Cell 18: 2493–2505. 17088607 10.1105/tpc.106.046136 PMC1626605

[nph20172-bib-0083] Patro R , Duggal G , Love MI , Irizarry RA , Kingsford C . 2017. Salmon provides fast and bias‐aware quantification of transcript expression. Nature Methods 14: 417–419. 28263959 10.1038/nmeth.4197 PMC5600148

[nph20172-bib-0084] Plackett ARG , Emms DM , Kelly S , Hetherington AM , Langdale JA . 2021. Conditional stomatal closure in a fern shares molecular features with flowering plant active stomatal responses. Current Biology 31: 4560–4570. 34450089 10.1016/j.cub.2021.08.008

[nph20172-bib-0085] Qi G‐N , Yao F‐Y , Ren H‐M , Sun S‐J , Tan Y‐Q , Zhang Z‐C , Qiu B‐S , Wang Y‐F . 2018. The S‐type anion channel ZmSLAC1 plays essential roles in stomatal closure by mediating nitrate efflux in maize. Plant and Cell Physiology 59: 614–623. 29390155 10.1093/pcp/pcy015

[nph20172-bib-0086] Qin X , Zeevaart JAD . 1999. The 9‐*cis*‐epoxycarotenoid cleavage reaction is the key regulatory step of abscisic acid biosynthesis in water‐stressed bean. Proceedings of the National Academy of Sciences, USA 96: 15354–15361. 10.1073/pnas.96.26.15354 PMC24823 10611388

[nph20172-bib-0087] Qiu J , Henderson SW , Tester M , Roy SJ , Gilliham M . 2016. SLAH1, a homologue of the slow type anion channel SLAC1, modulates shoot Cl^−^ accumulation and salt tolerance in *Arabidopsis thaliana* . Journal of Experimental Botany 67: 4495–4505. 27340232 10.1093/jxb/erw237 PMC4973733

[nph20172-bib-0088] Renzaglia KS , Villarreal JC , Piatkowski BT , Lucas JR , Merced A . 2017. Hornwort stomata: architecture and fate shared with 400 million year old fossil plants without leaves. Plant Physiology 174: 788–797. 28584065 10.1104/pp.17.00156 PMC5462037

[nph20172-bib-0089] Reynolds TL , Bewley JD . 1993. Characterization of protein synthetic changes in a desiccation‐tolerant fern, *Polypodium virginianum*. Comparison of the effects of drying, rehydration and abscisic acid. Journal of Experimental Botany 44: 921–928.

[nph20172-bib-0090] Rui Y , Xiao C , Yi H , Kandemir B , Wang JZ , Puri VM , Anderson CT . 2017. POLYGALACTURONASE INVOLVED IN EXPANSION3 functions in seedling development, rosette growth, and stomatal dynamics in *Arabidopsis thaliana* . Plant Cell 29: 2413–2432. 28974550 10.1105/tpc.17.00568 PMC5774581

[nph20172-bib-0091] Ruszala EM , Beerling DJ , Franks PJ , Chater C , Casson SA , Gray JE , Hetherington AM . 2011. Land plants acquired active stomatal control early in their evolutionary history. Current Biology 21: 1030–1035. 21658945 10.1016/j.cub.2011.04.044

[nph20172-bib-0092] Schäfer N , Maierhofer T , Herrmann J , Jørgensen ME , Lind C , von Meyer K , Lautner S , Fromm J , Felder M , Hetherington AM *et al*. 2018. A tandem amino acid residue motif in guard cell SLAC1 anion channel of grasses allows for the control of stomatal aperture by nitrate. Current Biology 28: 1370–1379. 29706511 10.1016/j.cub.2018.03.027

[nph20172-bib-0093] Scherzer S , Maierhofer T , Al‐Rasheid KAS , Geiger D , Hedrich R . 2012. Multiple calcium‐dependent kinases modulate ABA‐activated guard cell anion channels. Molecular Plant 5: 1409–1412. 22933711 10.1093/mp/sss084

[nph20172-bib-0094] Schindelin J , Arganda‐Carreras I , Frise E , Kaynig V , Longair M , Pietzsch T , Preibisch S , Rueden C , Saalfeld S , Schmid B *et al*. 2012. fiji: an open‐source platform for biological‐image analysis. Nature Methods 9: 676–682. 22743772 10.1038/nmeth.2019 PMC3855844

[nph20172-bib-0095] Shinozawa A , Otake R , Takezawa D , Umezawa T , Komatsu K , Tanaka K , Amagai A , Ishikawa S , Hara Y , Kamisugi Y *et al*. 2019. SnRK2 protein kinases represent an ancient system in plants for adaptation to a terrestrial environment. Communications Biology 2: 55. 30675528 10.1038/s42003-019-0281-1 PMC6340887

[nph20172-bib-0096] Stevens RA , Martin ES . 1977. New structure associated with stomatal complex of the fern *Polypodium vulgare* . Nature 265: 331–334.

[nph20172-bib-0097] Su D , Yang L , Shi X , Ma X , Zhou X , Hedges SB , Zhong B . 2021. Large‐scale phylogenomic analyses reveal the monophyly of bryophytes and neoproterozoic origin of land plants. Molecular Biology and Evolution 38: 3332–3344. 33871608 10.1093/molbev/msab106 PMC8321542

[nph20172-bib-0098] Sun Y , Harpazi B , Wijerathna‐Yapa A , Merilo E , de Vries J , Michaeli D , Gal M , Cuming AC , Kollist H , Mosquna A . 2019. A ligand‐independent origin of abscisic acid perception. Proceedings of the National Academy of Sciences, USA 116: 24892–24899. 10.1073/pnas.1914480116 PMC6900503 31744875

[nph20172-bib-0099] Sun Y , Qiao Z , Muchero W , Chen J‐G . 2020. Lectin receptor‐like kinases: the sensor and mediator at the plant cell surface. Frontiers in Plant Science 11: 596301. 33362827 10.3389/fpls.2020.596301 PMC7758398

[nph20172-bib-0100] Sussmilch FC , Brodribb TJ , McAdam SAM . 2017. What are the evolutionary origins of stomatal responses to abscisic acid in land plants? Journal of Integrative Plant Biology 59: 240–260. 28093875 10.1111/jipb.12523

[nph20172-bib-0101] Sussmilch FC , Roelfsema MRG , Hedrich R . 2019a. On the origins of osmotically‐driven stomatal movements. New Phytologist 222: 84–90. 30444541 10.1111/nph.15593

[nph20172-bib-0102] Sussmilch FC , Schultz J , Hedrich R , Roelfsema MRG . 2019b. Acquiring control: the evolution of stomatal signalling pathways. Trends in Plant Science 24: 342–351. 30797685 10.1016/j.tplants.2019.01.002

[nph20172-bib-0114] Talavera G , Castresana J . 2007. Improvement of phylogenies after removing divergent and ambiguously aligned blocks from protein sequence alignments. Systematic Biology 56(4): 564–577. 17654362 10.1080/10635150701472164

[nph20172-bib-0103] Tougane K , Komatsu K , Bhyan SB , Sakata Y , Ishizaki K , Yamato KT , Kohchi T , Takezawa D . 2010. Evolutionarily conserved regulatory mechanisms of abscisic acid signaling in land plants: characterization of *ABSCISIC ACID INSENSITIVE1*‐like type 2C protein phosphatase in the liverwort *Marchantia polymorpha* . Plant Physiology 152: 1529–1543. 20097789 10.1104/pp.110.153387 PMC2832234

[nph20172-bib-0104] Vahisalu T , Kollist H , Wang Y‐F , Nishimura N , Chan W‐Y , Valerio G , Lamminmäki A , Brosché M , Moldau H , Desikan R . 2008. SLAC1 is required for plant guard cell S‐type anion channel function in stomatal signalling. Nature 452: 487–491. 18305484 10.1038/nature06608 PMC2858982

[nph20172-bib-0105] VanBuren R , Wai CM , Zhang Q , Song X , Edger PP , Bryant D , Michael TP , Mockler TC , Bartels D . 2017. Seed desiccation mechanisms co‐opted for vegetative desiccation in the resurrection grass *Oropetium thomaeum* . Plant, Cell & Environment 40: 2292–2306. 10.1111/pce.13027 28730594

[nph20172-bib-0106] Voss LJ , McAdam SAM , Knoblauch M , Rathje JM , Brodribb TJ , Hedrich R , Roelfsema MRG . 2018. Guard cells in fern stomata are connected by plasmodesmata, but control cytosolic Ca^2+^ levels autonomously. New Phytologist 219: 206–215. 29655174 10.1111/nph.15153

[nph20172-bib-0107] Wang X , Chen S , Zhang H , Shi L , Cao F , Guo L , Xie Y , Wang T , Yan X , Dai S . 2010. Desiccation tolerance mechanism in resurrection fern‐ally *Selaginella tamariscina* revealed by physiological and proteomic analysis. Journal of Proteome Research 9: 6561–6577. 20923197 10.1021/pr100767k

[nph20172-bib-0108] Widholm JM . 1972. The use of fluorescein diacetate and phenosafranine for determining viability of cultured plant cells. Stain Technology 47: 189–194. 4113995 10.3109/10520297209116483

[nph20172-bib-0109] Xiao L , Yobi A , Koster KL , He Y , Oliver MJ . 2018. Desiccation tolerance in *Physcomitrella patens*: rate of dehydration and the involvement of endogenous abscisic acid (ABA). Plant, Cell & Environment 41: 275–284. 10.1111/pce.13096 29105792

[nph20172-bib-0110] Xu Z , Xin T , Bartels D , Li Y , Gu W , Yao H , Liu S , Yu H , Pu X , Zhou J *et al*. 2018. Genome analysis of the ancient tracheophyte *Selaginella tamariscina* reveals evolutionary features relevant to the acquisition of desiccation tolerance. Molecular Plant 11: 983–994. 29777775 10.1016/j.molp.2018.05.003

[nph20172-bib-0111] Yi H , Rui Y , Kandemir B , Wang JZ , Anderson CT , Puri VM . 2018. Mechanical effects of cellulose, xyloglucan, and pectins on stomatal guard cells of *Arabidopsis thaliana* . Frontiers in Plant Science 9: 1566. 30455709 10.3389/fpls.2018.01566 PMC6230562

[nph20172-bib-0112] Zhang J , Wang N , Miao Y , Hauser F , McCammon JA , Rappel W‐J , Schroeder JI . 2018. Identification of SLAC1 anion channel residues required for CO_2_/bicarbonate sensing and regulation of stomatal movements. Proceedings of the National Academy of Sciences, USA 115: 11129–11137. 10.1073/pnas.1807624115 PMC6217375 30301791

[nph20172-bib-0113] Zhang L , Li X , Li D , Sun Y , Li Y , Luo Q , Liu Z , Wang J , Li X , Zhang H *et al*. 2018. CARK1 mediates ABA signaling by phosphorylation of ABA receptors. Cell Discovery 4: 30. 29928509 10.1038/s41421-018-0029-y PMC6006248

