# Supplementary material for: Gaining or cutting SLAC: the evolution of plant guard cell signalling pathways
Source: New Phytol. 2024 Oct 6;244(6):2295–310. doi: 10.1111/nph.20172 (PMC11579433; doi:10.1111/nph.20172)
Supplement: Supplementary file 1 — Fig. S1 Most common annotated domains of differentially expressed orthogroups. Fig. S2 Relative expression of orthogroups containing abscisic acid biosynthesis pathway components. Fig. S3 Additional data for fern SLAC homologue activity and expression. Fig. S4 Phylogeny of the SLAC/SLAH family in streptophytes. Fig. S5 Phylogeny of the SnRK2 family in streptophytes. Fig. S6 Bimolecular fluorescence complementation experiments showing interaction between fern SLAC homologues and kinases tested in oocytes. Table S1 Overview of transcriptomes and differential gene expression analysis. Table S2 Genomic data used for gene expression analysis and evolutionary reconstruction. [file NPH-244-2295-s002.pdf]

***New Phytologist* Supporting Information**

Article title: Gaining or cutting SLAC: the evolution of plant guard cell signalling pathways
Authors: Frances C. Sussmilch, Tobias Maierhofer, Johannes Herrmann, Lena J. Voss, Christof Lind, Maxim Messerer, Heike M. Müller, Maria S. Bünner, Peter Ache, Klaus F. X. Mayer, Dirk Becker, M. Rob G. Roelfsema, Dietmar Geiger, Jörg Schultz, Rainer Hedrich
Article acceptance date: 08 September 2024

The following Supporting Information is available for this article:

**Fig. S1 Most common annotated domains of differentially expressed orthogroups.**

**Fig. S2 Relative expression of orthogroups containing abscisic acid (ABA) biosynthesis pathway components.**

**Fig. S3 Additional data for fern SLAC homolog activity and expression**.

**Fig. S4 Phylogeny of the SLAC/SLAH family in streptophytes.**

**Fig. S5 Phylogeny of the SnRK2 family in streptophytes.**

**Fig. S6 Bimolecular fluorescence complementation (BiFC) experiments showing interaction between fern SLAC homologs and kinases tested in oocytes**.

**Table S1 Overview of transcriptomes and differential gene expression analysis.**

**Table S2 Genomic data used for gene expression analysis and evolutionary reconstruction.**

**Table S3 Complete list of differentially expressed (DE) orthogroups from Figure 1 (separate excel file).**

**Table S4 Gene sequence details (separate excel file).**

**Table S5 Primer details.**

**Table S6 Orthogroup details for genes of interest for Figure 2 (separate excel file).**

**Fig. S1 Most common annotated domains of differentially expressed orthogroups.** The top 15 most common annotated domains of orthogroups up- or down-regulated in guard cell relative to whole leaf samples in all angiosperm (*Arabidopsis thaliana*, *Hordeum vulgare*) and fern (*Ceratopteris richardii*, *Polypodium vulgare*) species examined ('shared'; black), both angiosperm but neither fern species ('angiosperms'; grey), and both fern but neither angiosperm ('ferns'; white). Counts are normalised to the total number of genes per orthogroup. Abbreviations are as follows:. [1] – "(a.k.a. RRM, RBD, or RNP domain)", ...[2] – "associated with various cellular activities (AAA)". See Table S3 for all annotated domains related to these orthogroups.

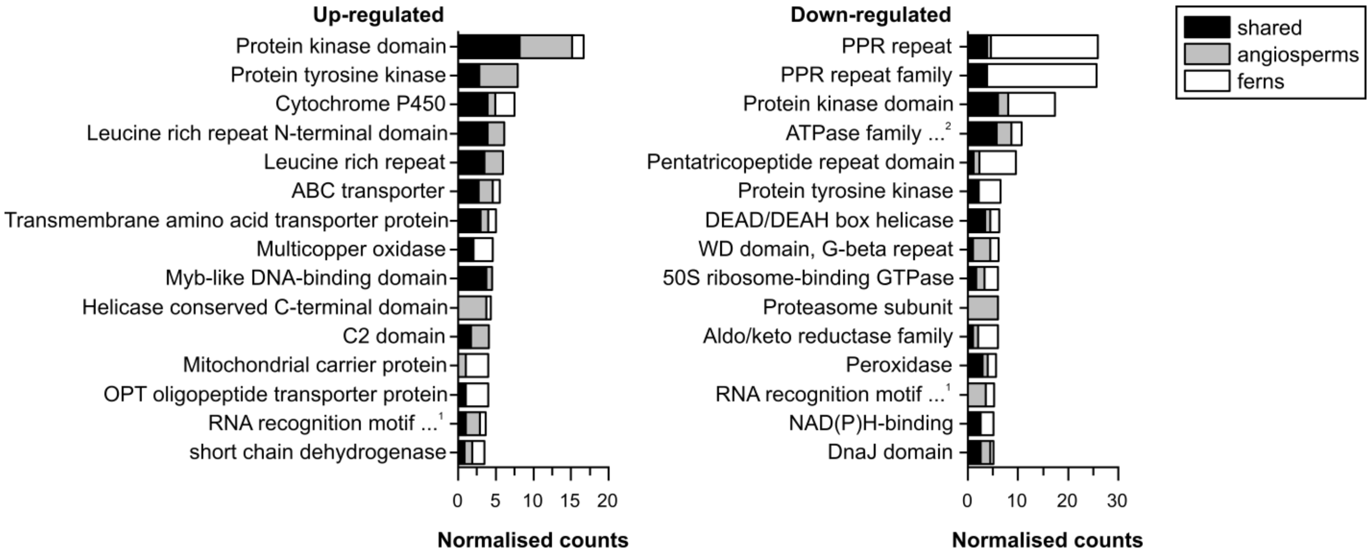

**Fig. S2 Relative expression of orthogroups containing abscisic acid (ABA) biosynthesis pathway components.** The core Arabidopsis ABA biosynthesis pathway is shown on the left with ABA precursors shown interconnected by arrows, and the proteins that catalyse each step indicated. Orthogroups/subclades containing the genes that encode these proteins are shown together in boxes on the right with each circle representing a different gene and colour coding representing relative expression in guard cell-enriched samples compared to whole leaves is shown for angiosperm (At, *Arabidopsis thaliana*; Hv, *Hordeum vulgare* barley) and fern (Cr, *Ceratopteris richardii*; Pv, *Polypodium vulgare*) models as indicated. For *P. vulgare* samples only, whole leaf vs 'leaf samples without abaxial epidermis' (thus guard cells removed) were also included and used to separate guard cell-enriched genes with a higher level of stringency (red; "enriched both comparisons" = expression higher in guard cells than leaves, and higher in whole leaves than leaves without guard cells). Please note that the ABA2 clade of SDRs is only found in angiosperms (Moummou *et al.*, 2012; Sussmilch *et al.*, 2017), but based on the leaky nature of *aba2* mutants (Cheng *et al.*, 2002; McAdam *et al.*, 2015), other related SDRs (e.g. within the same SDR110C clade) are likely to be able to catalyse this step of ABA biosynthesis; Arabidopsis and barley ABA2 orthologs (left; determined phylogenetically) are shown separately to other SDR110C clade members for clarity. See Table S6 for orthogroup details.

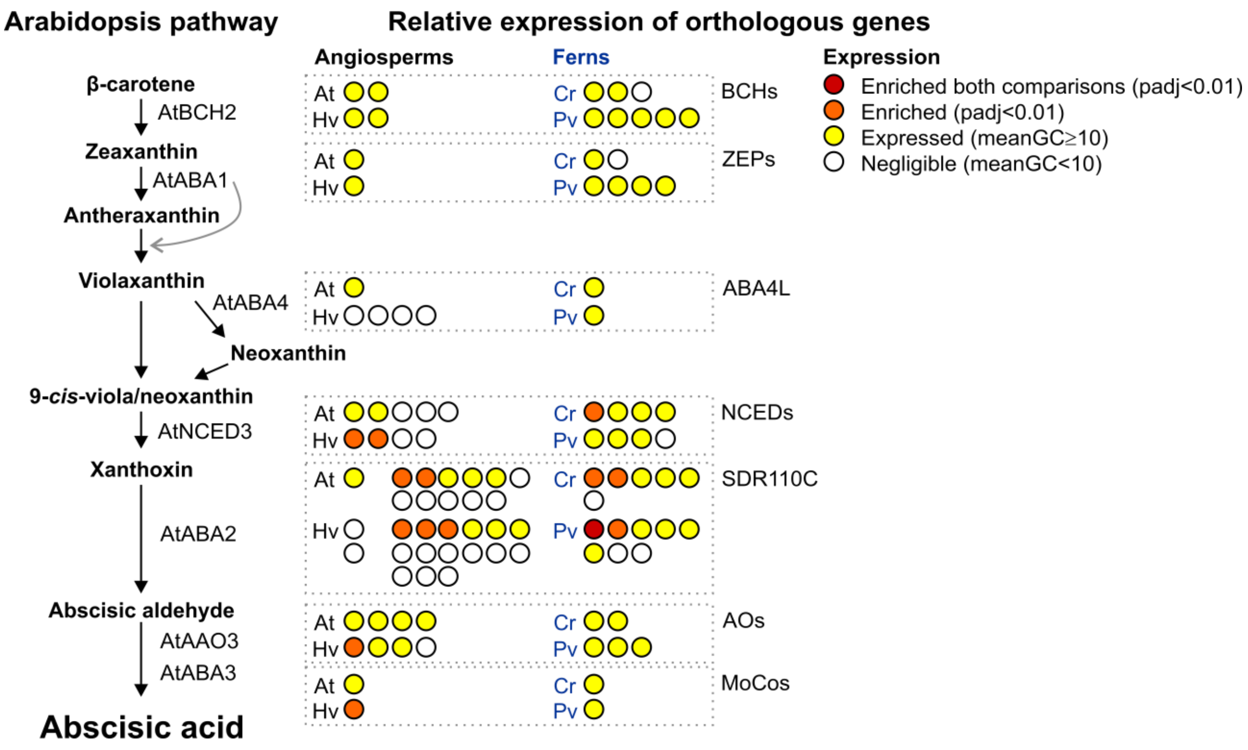

**Fig. S3 Additional data for fern SLAC homolog activity and expression**. **(a-c)** Mean whole-oocyte current measurements at −100 mV in nitrate-based solution of **(a)** wild-type SLAC homologs from the angiosperm *Arabidopsis thaliana* and the fern *Ceratopteris richardii*, **(b)** PvSLAC1a V663L in which the C-terminal motif (Lind *et al.*, 2015) of the *Polypodium vulgare* protein is altered to match Arabidopsis, and (**c**) a gate mutant of CrSLAC1a, co-expressed with or without kinases in *Xenopus* oocytes (mean + SEM, n ≥ 3). **(d)** Expression of *P. vulgare* and *C. richardii* genes of interest in whole leaf and guard cell-enriched samples (mean + SEM, n ≥ 3). Please note that *CrSLAC1f* was not represented in our sporophyte transcriptome. Counts were normalised by sample-specific size factors determined by median ratio of gene counts relative to geometric mean per gene.

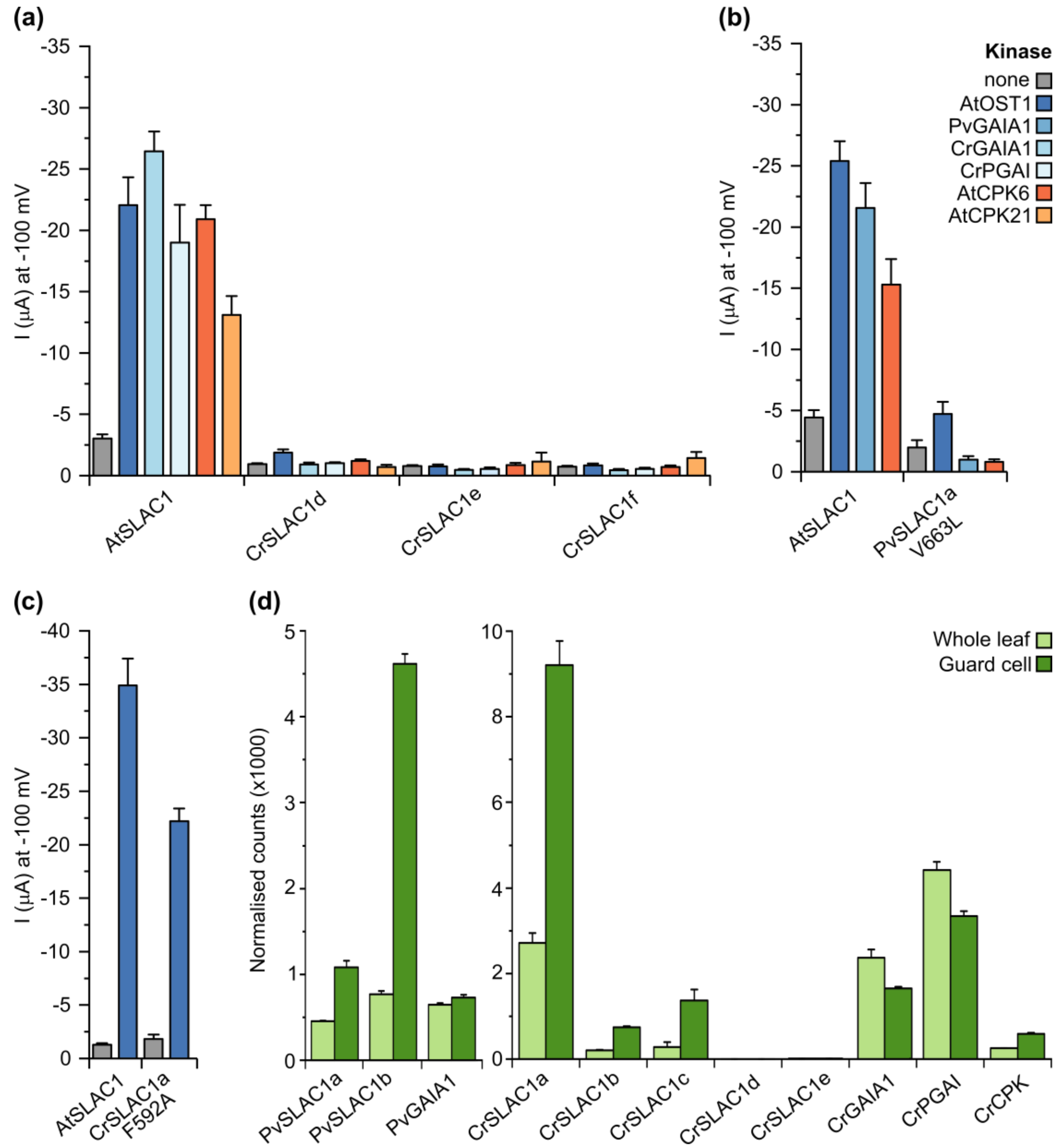

**Fig. S4 Phylogeny of the SLAC/SLAH family in streptophytes.** SLAC/SLAH channels measured in *Xenopus* oocytes in this study are shown in bold. Bootstrap values from 1000 replicates are shown as percentages on nodes of the maximum likelihood phylogenetic tree. Full sequence and species details are given in Table S4.

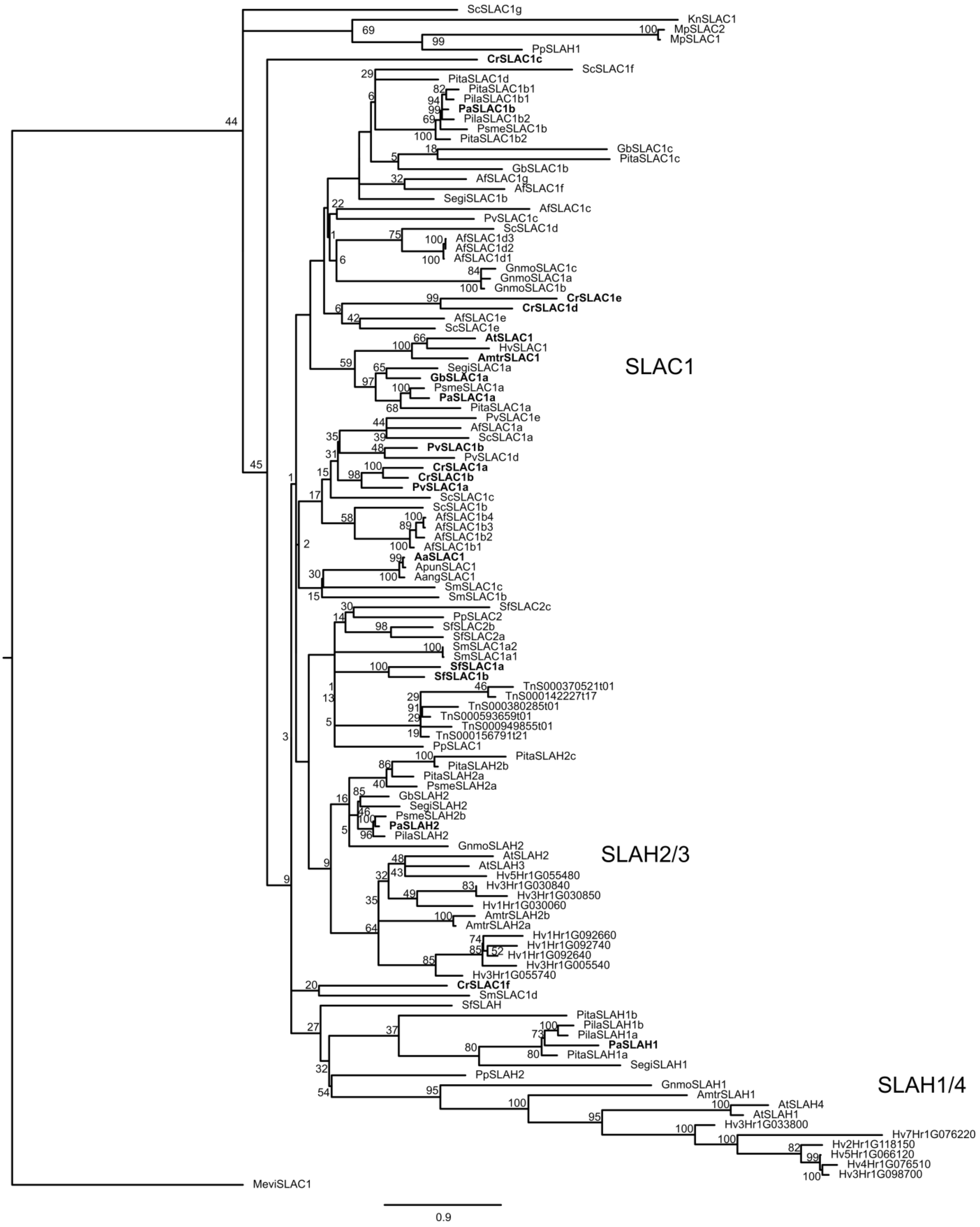

**Fig. S5 Phylogeny of the SnRK2 family in streptophytes.** Kinases from the OST1 subclade measured in *Xenopus* oocytes in this study are shown in bold. Bootstrap values from 1000 replicates are shown as percentages on nodes of the maximum likelihood phylogenetic tree. Full sequence and species details are given in Table S4.

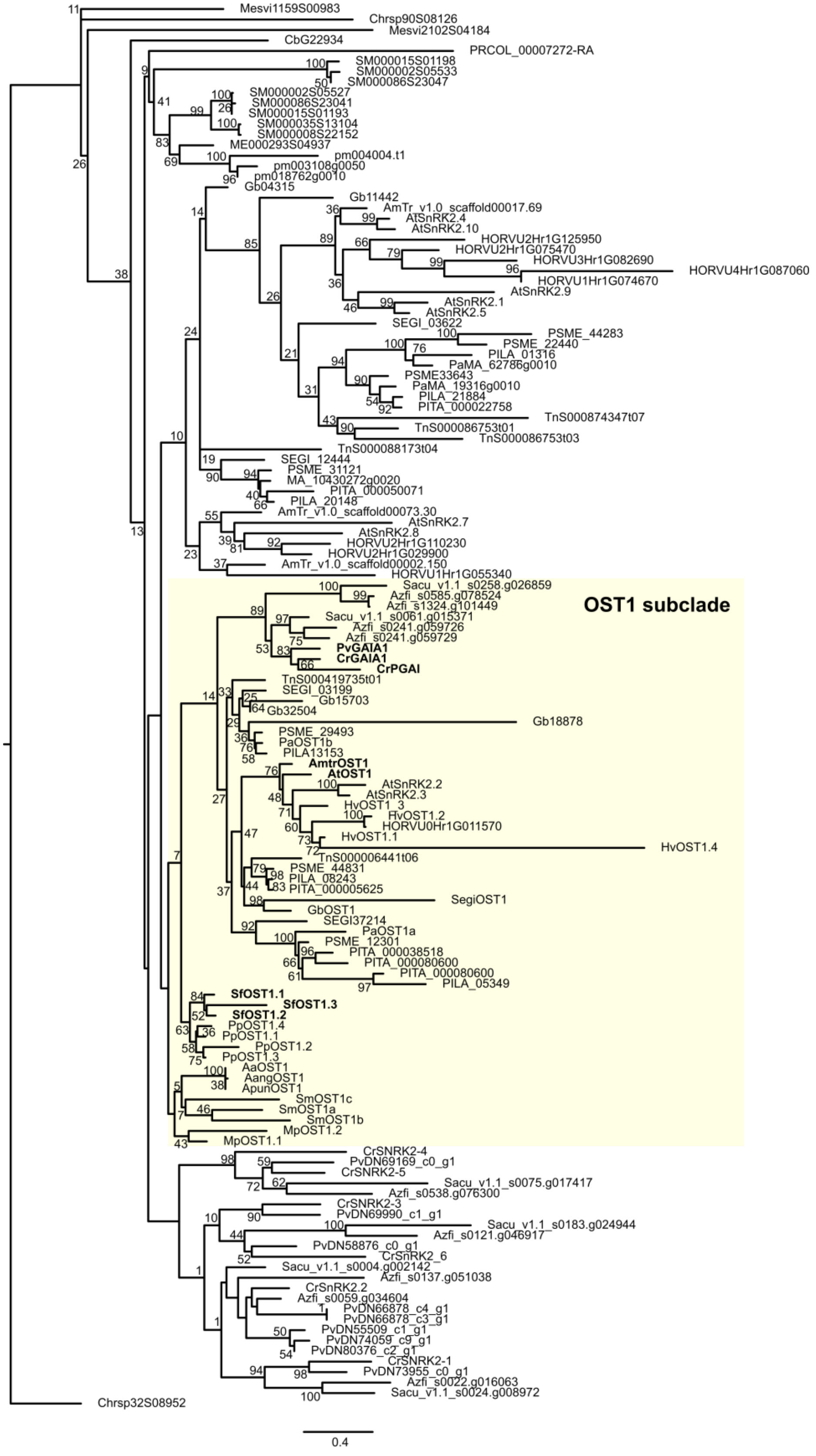

**Fig. S6 Bimolecular fluorescence complementation (BiFC) experiments showing interaction between fern SLAC homologs and kinases tested in oocytes**. Kinases were fused to the N-terminal half of a YFP molecule ($YFP^{NT}$) while the anion channels were fused to the C-terminal half of the YFP ($YFP^{CT}$). A quarter of a representative oocyte is shown for each interaction.

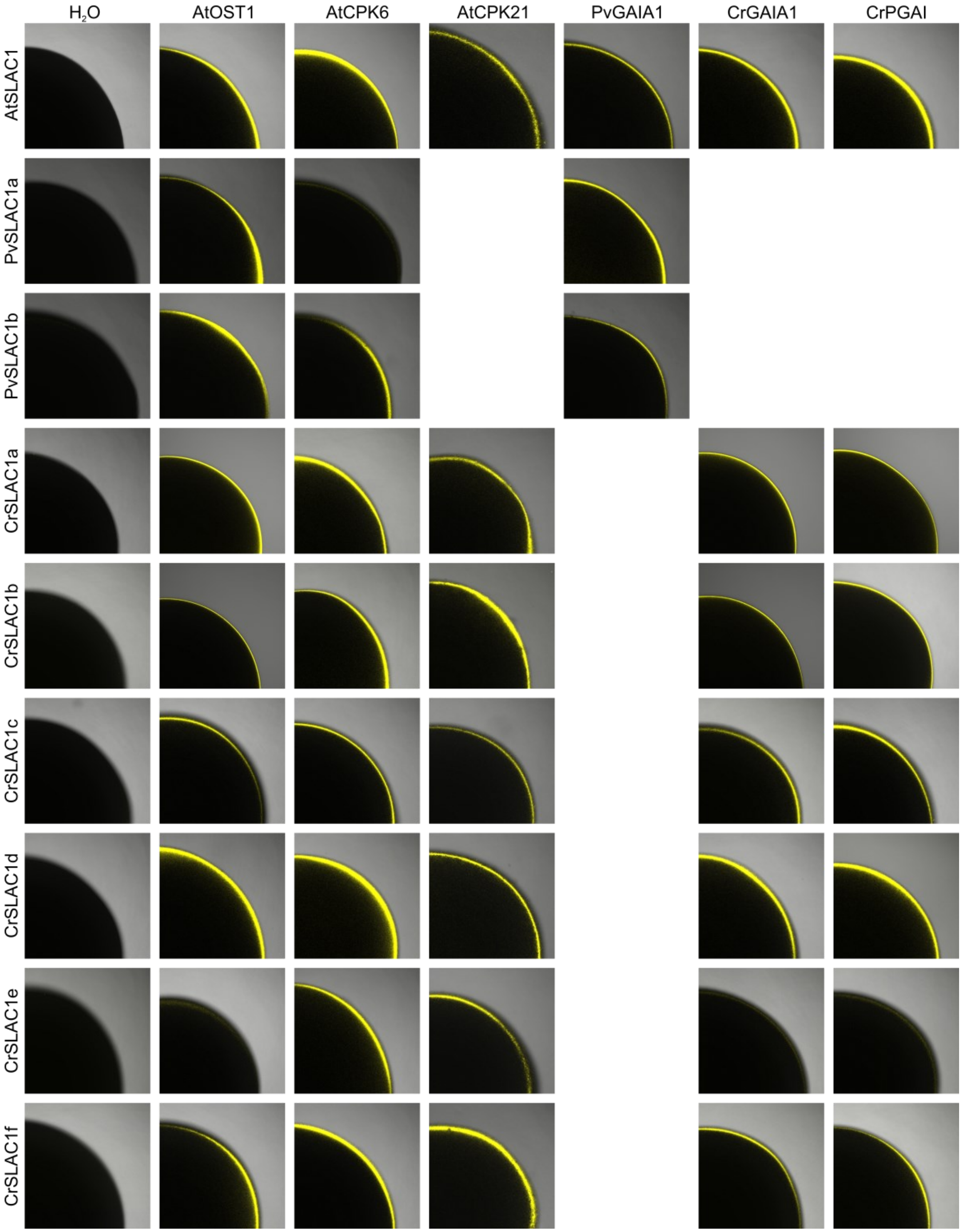

**Table S1 Overview of transcriptomes and differential gene expression analysis.** Significance cutoff for guard cell genes: padj <= 0.01 and logfold < 0.

| Species | Unigenes | Isoforms | Protein coding (Uni)genes | Guard cell protein coding genes |
|---|---|---|---|---|
| *Polypodium vulgare* | 293847 | 635910 | 50833 | 8333 |
| *Ceratopteris richardii* | 165371 | 347757 | 30260 | 1900 |
| *Arabidopsis thaliana* | | | 27628 | 1826 |
| *Hordeum vulgare* | | | 37673 | 6653 |

**Table S2 Genomic data used for gene expression analysis and evolutionary reconstruction.**

| Species | Data sets | References |
|---|---|---|
| *Amborella trichopoda* | Atrichopoda_291_v1.0.protein.fa.gz | Albert *et al.* (2013) |
| *Arabidopsis thaliana* | Genome: Arabidopsis_thaliana.TAIR10.dna.toplevel.fa.gz<br>Proteome: Arabidopsis_thaliana.TAIR10.pep.all.fa | Lamesch *et al.* (2012) |
| *Chara braunii* | mRNA_Chbra_active_pep_20170414.tfa.xz | Nishiyama *et al.* (2018) |
| *Ginkgo biloba* | Gb.pep.fa | Guan *et al.* (2016) |
| *Hordeum vulgare* | Genome: Hordeum_vulgare.IBSC_v2.dna.toplevel.fa.gz<br>Proteome: Hordeum_vulgare.Hv_IBSC_PGSB_v2.pep.all.fa | Mascher *et al.* (2017) |
| *Klebsormidium nitens* | 160614_klebsormidium_v1.1_AA.fasta | Hori *et al.* (2014) |
| *Marchantia polymorpha* | Mpolymorpha_320_v3.1.protein.fa.gz | Bowman *et al.* (2017) |
| *Picea abies* | Pabies1.0-all-pep.faa.gz | Nystedt *et al.* (2013) |
| *Physcomitrium/ Physcomitrella patens* | Ppatens_318_v3.3.protein.fa.gz | Lang *et al.* (2018) |
| *Salvinia cucullata* | Salvinia_cucullata.protein.highconfidence_v1.2.fasta | Li *et al.* (2018) |
| *Selaginella moellendorffii* | Smoellendorffii_91_v1.0.protein.fa.gz | Banks *et al.* (2011) |

**Table S5 Primer details.** See Table S4 for all gene sequence details.

| Gene | Purpose | Primer sequences (5' to 3') |
|---|---|---|
| *AaSLAC1* | Cloning full length CDS without stop codon | F:GGCTTAAUATGAGCAGTAATAGGCCGGCGGGGTC |
| | | R1:GGTTTAAUCCTACGCCGTTTGAAGTGACG |
| | Cloning full length CDS with stop codon | F:GGCTTAAUATGAGCAGTAATAGGCCGGCGGGGTC |
| | | R2:GGTTTAAUTTATACGCCGTTTGAAGTGA |
| *AmtrOST1* | Cloning full length CDS without stop codon | F:GGCTTAAUATGGATCGGACTGCTCTCAC |
| | | R1:GGTTTAAUCCCATTGCATAGACGATCTCACCAC |
| | Cloning full length CDS with stop codon | F:GGCTTAAUATGGATCGGACTGCTCTCAC |
| | | R2:GGTTTAAUTTACATTGCATAGACGATCTCACC |
| *AmtrSLAC1* | Cloning full length CDS without stop codon | F1:GGCTTAAUATGAAGCCAGATAAATATTGCAG |
| | | R1:GGTTTAAUCCGACCTTTCCTCCACTTGCAT |
| | Cloning full length CDS with stop codon | F1:GGCTTAAUATGAAGCCAGATAAATATTGCAG |
| | | R2:GGTTTAAUTCAGACCTTTCCTCCACTTG |
| *CrCPK* | Cloning full length CDS without stop codon | F:GGCTTAAUATGGGTAACTGCTGCAGCAA |
| | | R1:GGTTTAAUCCCTTCTGTAGTGAGCCATCTTTC |
| | Cloning full length CDS with stop codon | F:GGCTTAAUATGGGTAACTGCTGCAGCAA |
| | | R2:GGTTTAAUCTACTTCTGTAGTGAGCCATCTT |
| *CrSLAC1c* | Cloning full length CDS without stop codon | F:GGCTTAAUATGGCAAGAGAAGATTGCAAAG |
| | | R1:GGTTTAAUCCGCCTTGGGTCCATGCGAGCTG |
| | Cloning full length CDS with stop codon | F:GGCTTAAUATGGCAAGAGAAGATTGCAAAG |
| | | R2:GGTTTAAUTTAGCCTTGGGTCCATGC |
| *CrSLAC1d* | Cloning full length CDS without stop codon | F:GGCTTAAUATGCCTTCATCAGCGCGTTC |
| | | R1:GGTTTAAUCCTACTGGTAGGTCATTTTCCT |
| | Cloning full length CDS with stop codon | F:GGCTTAAUATGCCTTCATCAGCGCGTTC |
| | | R2:GGTTTAAUTCATACTGGTAGGTCATTTTCCT |
| *CrSLAC1e* | Cloning full length CDS without stop codon | F:GGCTTAAUATGGGATATGTATCCAATATTGAAG |
| | | R1:GGTTTAAUCCGATATAACATGGTAGAAAATCTTT |
| | Cloning full length CDS with stop codon | F:GGCTTAAUATGGGATATGTATCCAATATTGAAG |
| | | R2:GGTTTAAUTTAGATATAACATGGTAGAAAATC |
| *CrSLAC1f* | Cloning full length CDS without stop codon | F:GGCTTAAUATGTATCCATACATGAAGGA |
| | | R1:GGTTTAAUCCTTTTGCGCCCCTCTGTGA |
| | Cloning full length CDS with stop codon | F:GGCTTAAUATGTATCCATACATGAAGGA |
| | | R2:GGTTTAAUCTATTTTGCGCCCCTCTG |
| *GbSLAC1a* | Cloning full length CDS without stop codon | F:GGCTTAAUATGGACACCAAAATCGAAAAA |
| | | R1:GGTTTAAUCCTCTTGTTTGTTTCGTAAAGC |
| | Cloning full length CDS with stop codon | F:GGCTTAAUATGGACACCAAAATCGAAAAA |
| | | R2:GGTTTAAUTTATCTTGTTTGTTTCGTAAAGC |
| *PaSLAC1a* | Cloning full length CDS without stop codon | F1:GGCTTAAUATGAATCCCATAAGACCTGCAA |
| | | R1:GGTTTAAUCCTGGCAATTTATGGTTGAAGAGA |
| | Cloning full length CDS with stop codon | F1:GGCTTAAUATGAATCCCATAAGACCTGCAA |
| | | R2:GGTTTAAUTCATGGCAATTTATGGTTGAA |
| *PaSLAC1b* | Cloning full length CDS without stop codon | F:GGCTTAAUATGGAAAACCAGAACTCTTTCA |
| | | R1:GGTTTAAUCCATCTAGGGGACCATGACCAG |
| | Cloning full length CDS with stop codon | F:GGCTTAAUATGGAAAACCAGAACTCTTTCA |
| | | R2:GGTTTAAUTTAATCTAGGGGACCATGACCA |
| *PaSLAH1* | Cloning full length CDS without stop codon | F:GGCTTAAUATGTCGCAGATAAGCATGAGC |
| | | R1:GGTTTAAUCCCTGAAAATTAGCTGGTGGGTTT |
| | Cloning full length CDS with stop codon | F:GGCTTAAUATGTCGCAGATAAGCATGAGC |
| | | R2:GGTTTAAUTCACTGAAAATTAGCTGGTG |
| *PaSLAH2* | Cloning full length CDS without stop codon | F:GGCTTAAUATGGAGTCAATTGAAATCACCA |
| | | R1:GGTTTAAUCCGAGAGATAATTCAGAGAGTAAAGT |
| | Cloning full length CDS with stop codon | F:GGCTTAAUATGGAGTCAATTGAAATCACCA |
| | | R2:GGTTTAAUTTAGAGAGATAATTCAGAGAGTAAA |
| *PvGAIA1* | Cloning full length CDS without stop codon | F:GGCTTAAUATGGATCGCGTTGTTGCGGGTGCT |
| | | R1:GGTTTAAUCCAATAGCACTCACATACTCTCCA |
| | Cloning full length CDS with stop codon | F:GGCTTAAUATGGATCGCGTTGTTGCGGGTGCT |
| | | R2:GGTTTAAUTCAAATAGCACTCACATACTCTC |

| Gene | Purpose | Primer sequences (5' to 3') |
|---|---|---|
| *PvSLAC1a* | Cloning full length CDS without stop codon | F1:GGCTTAAUATGGCAACATATGGTGCGC |
| | | R1:GGTTTAAUCCTGCATCTCTGTCGATAGAATTTTCC |
| | Cloning full length CDS with stop codon | F1:GGCTTAAUATGGCAACATATGGTGCGC |
| | | R2:GGCTTAAUTCATGCATCTCTGTCGATAGAATTTT |
| | Introducing V663L mutation | F2:ATcTCGCCAUTGCCATAACAAGGAAGAAGCA+R1/R2, |
| | | F1+R3:ATGGCGAgAUCATTCGGAAACAAACTGCCC |
| *PvSLAC1b* | Cloning full length CDS without stop codon | F:GGCTTAAUATGCGAAACAACGGTGCA |
| | | R1:GGTTTAAUCCTGCTACACCCTCTTGAAGC |
| | Cloning full length CDS with stop codon | F:GGCTTAAUATGCGAAACAACGGTGCA |
| | | R2:GGTTTAAUTCATGCTACACCCTCTTGAA |
| *SfOST1-1* | Cloning full length CDS without stop codon | F:GGCTTAAUATGGACCCCTTGGAGATCA |
| | | R1:GGTTTAAUCCTATAGCGCACACAAACTCCCCACT |
| | Cloning full length CDS with stop codon | F:GGCTTAAUATGGACCCCTTGGAGATCA |
| | | R2:GGTTTAAUTTATATAGCGCACACAAACTCC |
| *SfOST1-2* | Cloning full length CDS without stop codon | F:GGCTTAAUATGGACTTTTCGAGTATACAAGATGT |
| | | R1:GGTTTAAUCCTATGGCACACACAAATTCCCCACT |
| | Cloning full length CDS with stop codon | F:GGCTTAAUATGGACTTTTCGAGTATACAAGATGT |
| | | R2:GGTTTAAUTTATATGGCACACACAAATTCC |
| *SfOST1-3* | Cloning full length CDS without stop codon | F:GGCTTAAUATGGATTTGTTTTCTGGGATA |
| | | R1:GGTTTAAUCCTATAGCACACACAAATTCCCCACT |
| | Cloning full length CDS with stop codon | F:GGCTTAAUATGGATTTGTTTTCTGGGATA |
| | | R2:GGTTTAAUTTATATAGCACACACAAATTCCCCACT |
| *SfSLAC1a* | Cloning full length CDS without stop codon | F:GGCTTAAUATGGACTCTGCTGTTGCTGTT |
| | | R1:GGTTTAAUCCAGCTTCAAGAGGCTGATCAGGAAG |
| | Cloning full length CDS with stop codon | F:GGCTTAAUATGGACTCTGCTGTTGCTGTT |
| | | R2:GGTTTAAUTCAAGCTTCAAGAGGCTGAT |
| *SfSLAC1b* | Cloning full length CDS without stop codon | F:GGCTTAAUATGGCAGCAGAGTTGCGACGGGAGGT |
| | | R1:GGTTTAAUCCACCTCTAGAAGAGGTCACAC |
| | Cloning full length CDS with stop codon | F:GGCTTAAUATGGCAGCAGAGTTGCGACGGGAGGT |
| | | R2:GGTTTAAUTATGCGCTGCTGACAACTTT |

**Supplemental References**


**Albert VA, Barbazuk WB, dePamphilis CW, Der JP, Leebens-Mack J, Ma H, Palmer JD, Rounsley S, Sankoff D, Schuster SC, et al. 2013.** The *Amborella* genome and the evolution of flowering plants. *Science* **342**(6165): 1241089.

**Banks JA, Nishiyama T, Hasebe M, Bowman JL, Gribskov M, dePamphilis C, Albert VA, Aono N, Aoyama T, Ambrose BA, et al. 2011.** The *Selaginella* genome identifies genetic changes associated with the evolution of vascular plants. *Science* **332**(6032): 960-963.

**Bowman JL, Kohchi T, Yamato KT, Jenkins J, Shu S, Ishizaki K, Yamaoka S, Nishihama R, Nakamura Y, Berger F, et al. 2017.** Insights into land plant evolution garnered from the *Marchantia polymorpha* genome. *Cell* **171**(2): 287-304.e215.

**Cheng W-H, Endo A, Zhou L, Penney J, Chen H-C, Arroyo A, Leon P, Nambara E, Asami T, Seo M, et al. 2002.** A unique short-chain dehydrogenase/reductase in Arabidopsis glucose signaling and abscisic acid biosynthesis and functions. *The Plant Cell* **14**(11): 2723-2743.

**Guan R, Zhao Y, Zhang H, Fan G, Liu X, Zhou W, Shi C, Wang J, Liu W, Liang X, et al. 2016.** Draft genome of the living fossil *Ginkgo biloba*. *GigaScience* **5**(1): 49.

**Hori K, Maruyama F, Fujisawa T, Togashi T, Yamamoto N, Seo M, Sato S, Yamada T, Mori H, Tajima N, et al. 2014.** *Klebsormidium flaccidum* genome reveals primary factors for plant terrestrial adaptation. *Nature Communications* **5**: 3978.

**Lamesch P, Berardini TZ, Li D, Swarbreck D, Wilks C, Sasidharan R, Muller R, Dreher K, Alexander DL, Garcia-Hernandez M, et al. 2012.** The Arabidopsis Information Resource (TAIR): improved gene annotation and new tools. *Nucleic Acids Research* **40**(D1): D1202-D1210.

**Lang D, Ullrich KK, Murat F, Fuchs J, Jenkins J, Haas FB, Piednoel M, Gundlach H, Van Bel M, Meyberg R, et al. 2018.** The *Physcomitrella patens* chromosome-scale assembly reveals moss genome structure and evolution. *The Plant Journal* **93**(3): 515–533.

**Li F-W, Brouwer P, Carretero-Paulet L, Cheng S, de Vries J, Delaux P-M, Eily A, Koppers N, Kuo L-Y, Li Z, et al. 2018.** Fern genomes elucidate land plant evolution and cyanobacterial symbioses. *Nature Plants* **4**(7): 460-472.

**Lind C, Dreyer I, López-Sanjurjo EJ, von Meyer K, Ishizaki K, Kohchi T, Lang D, Zhao Y, Kreuzer I, Al-Rasheid KAS, et al. 2015.** Stomatal guard cells co-opted an ancient ABA-dependent desiccation survival system to regulate stomatal closure. *Current Biology* **25**(7): 928-935.

**Mascher M, Gundlach H, Himmelbach A, Beier S, Twardziok SO, Wicker T, Radchuk V, Dockter C, Hedley PE, Russell J, et al. 2017.** A chromosome conformation capture ordered sequence of the barley genome. *Nature* **544**: 427-433.

**McAdam SAM, Sussmilch FC, Brodribb TJ, Ross JJ. 2015.** Molecular characterization of a mutation affecting abscisic acid biosynthesis and consequently stomatal responses to humidity in an agriculturally important species. *AoB Plants* **7**: plv091.

**Moummou H, Kallberg Y, Tonfack LB, Persson B, van der Rest B. 2012.** The plant Short-Chain Dehydrogenase (SDR) superfamily: genome-wide inventory and diversification patterns. *BMC Plant Biology* **12**(1): 1-17.

**Nishiyama T, Sakayama H, de Vries J, Buschmann H, Saint-Marcoux D, Ullrich KK, Haas FB, Vanderstraeten L, Becker D, Lang D, et al. 2018.** The *Chara* genome: secondary complexity and implications for plant terrestrialization. *Cell* **174**(2): 448-464.e424.

**Nystedt B, Street NR, Wetterbom A, Zuccolo A, Lin Y-C, Scofield DG, Vezzi F, Delhomme N, Giacomello S, Alexeyenko A, et al. 2013.** The Norway spruce genome sequence and conifer genome evolution. *Nature* **497**(7451): 579-584.

**Sussmilch FC, Brodribb TJ, McAdam SAM. 2017.** What are the evolutionary origins of stomatal responses to abscisic acid in land plants? *Journal of Integrative Plant Biology* **59**(4): 240-260.
